# Supplementary material for: A bioabsorbable body-coupling-electrotherapy suture
Source: Nat Commun. 2025 Dec 10;16:11156. doi: 10.1038/s41467-025-66045-8 (PMC12708654; doi:10.1038/s41467-025-66045-8)
Supplement: Supplementary file 1 — Supplementary Information [file 41467_2025_66045_MOESM1_ESM.pdf]

Supporting Information

A bioabsorbable body-coupling-electrotherapy suture

Zhouquan Sun[1,#], Yuefan Jin[2,#], Hui Su[1,#], Yaogang Li[1], Qinghong Zhang[1], Kerui Li[1], Hongzhi Wang[1], Linpeng Li[2*], Shan-kai Yin[2], Chengyi Hou[1,2*], Hui Wang[2*]

Supplementary Figures

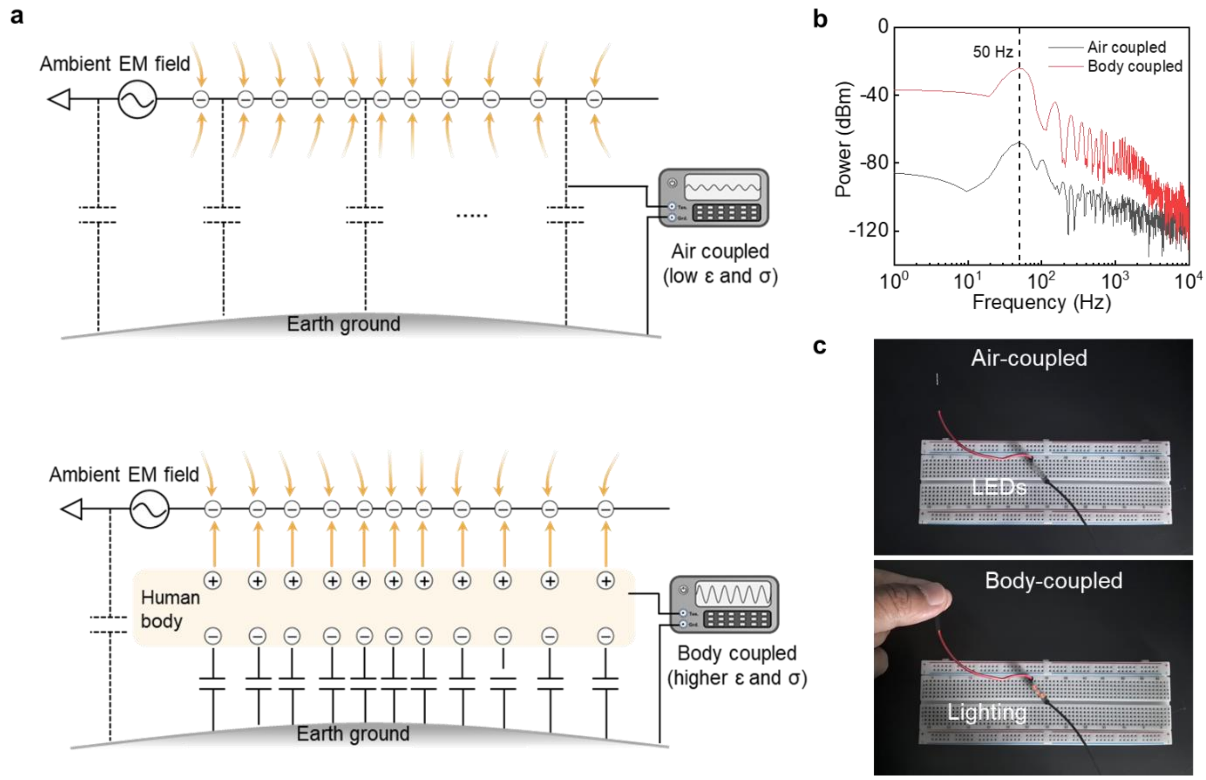

Figure S1. Circuit models of body-coupled EM energy sources. (a) Distribution of unipolar E-field in open space. Since air has a low dielectric constant (ε) and conductivity (σ), most of the energy is dissipated in the air environment, and its equivalent capacitance is the parallel connection of multiple parasitic capacitances (top). When the human body (high ε and σ) is in the EM, it induces a quasi-static polarization of the body. The EM field, the body and the earth together form a closed energy loop (bottom). (b) Maximum power spectrum of ambient EM waves coupled by the human body and air in various conventional environments, including offices, laboratories, outdoors, factories. (c) In contrast to air coupling, body-coupled EM energy directly lighting up three LEDs connected in series.

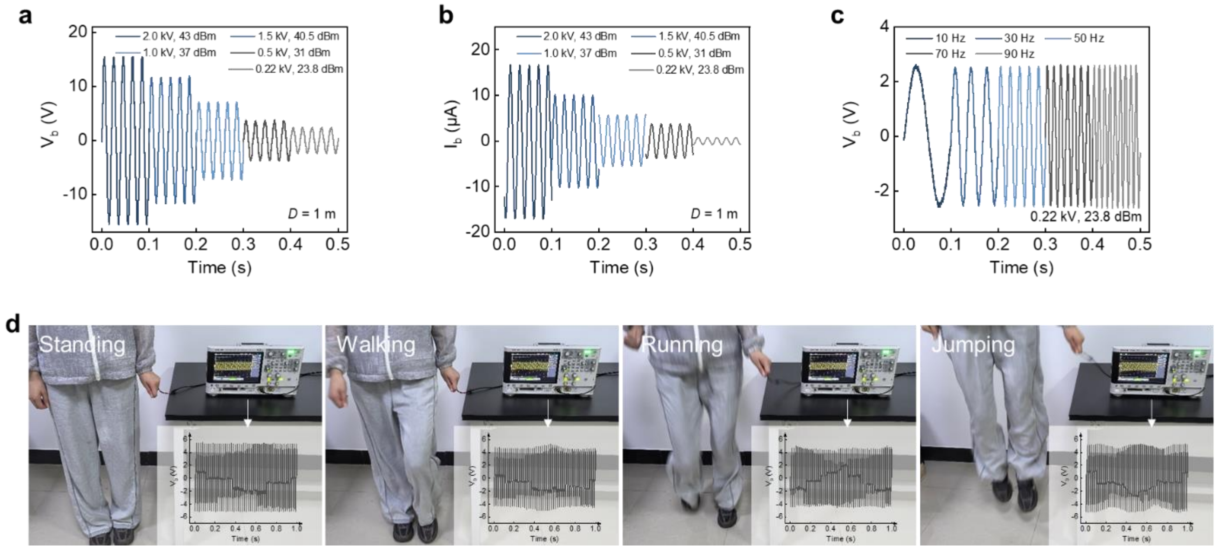

Figure S2. Electrical output of the body-coupled environment EM energy. The variation trend of (a) body-coupled voltage (V_b) and (b) current (I_b) under different environmental EM field intensities. (c) The V_b remains stable when the frequency of the environmental EM field varies within the frequency range (10-90 Hz) of the bioelectric signal. (d) Real-time voltage output of body-coupled EM energy in different movement states, including standing, walking, running, and jumping.

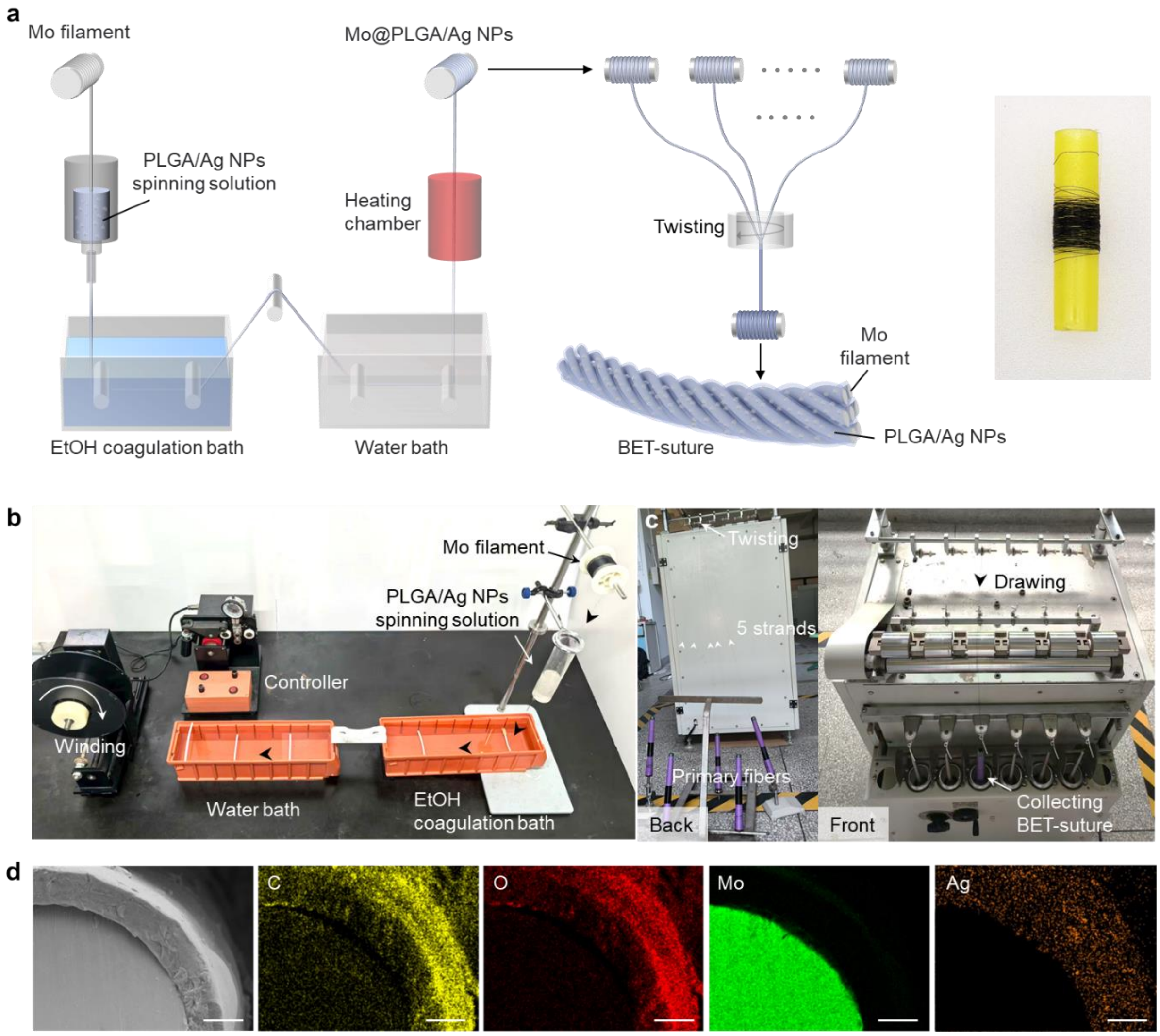

Figure S3. Schematic diagram of the continuous preparation of BET-suture and elemental distribution of the cross section. (a) Primary fiber (Mo@PLGA/Ag NPs) was obtained by coating PLGA/Ag NPs dielectric layer on Mo filament using modified wet-method technique. BET-suture was obtained by twisting the primary fibres.The physical picture shows a sample roll of BET-suture. (b) Physical illustration of the preparation of primary fiber (Mo@PLGA/Ag NPs) using the constructed wet-coating device. (c) Process of preparing BET-suture by twisting the primary fibers using a doubling and twisting combined testing machine. (d) Cross-sectional SEM and corresponding elemental mapping images of BET-suture. Scale bar: 15 μm.

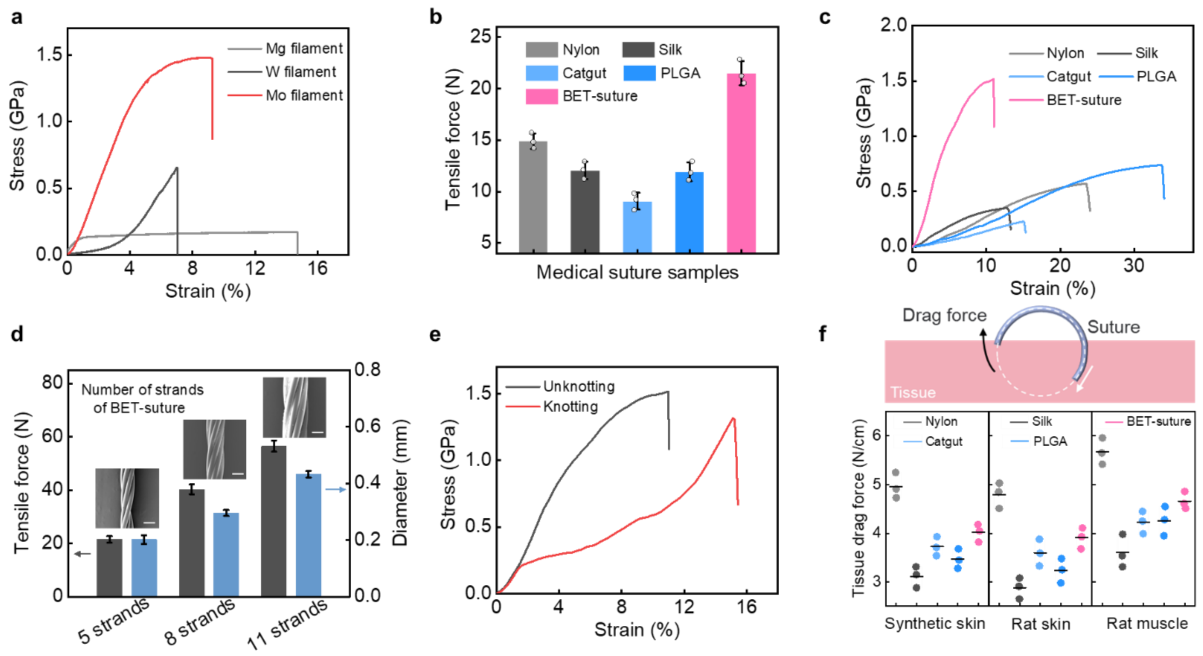

Figure S4. Mechanical properties and stitching characteristics of BET-suture. (a) Stress-strain curves of BET-suture prepared using Mo filament, W filament, and Mg filament. (b) Breaking tensile force and (c) stress-strain curves of different medical sutures. n = 3 independent samples. (d) Breaking tensile force (grey) and diameter (blue) of BET-suture twisted from 5, 8, and 11 strands of primary fibers, respectively. n = 3 independent samples. Scale bar: 200 µm. (e) Stress-strain curves of BET-suture before and after knotting. (f) Schematic diagram of the drag force test required for the suture to thread through the tissue and comparison of tissue drag force of different medical sutures in different tissue models. n = 3 independent samples. Data represent mean ± standard deviation.

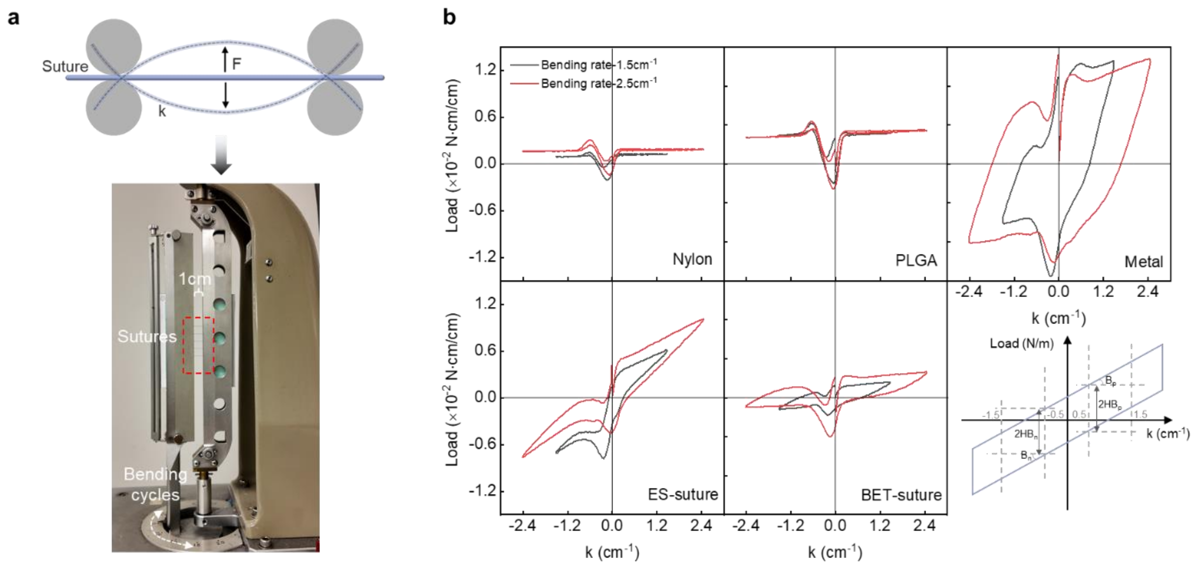

Figure S5. Bending properties of BET-suture. (a) Schematic and physical photos of the Kawabata Evaluation System for testing the bending stiffness of sutures. (b) Bending characteristic curves of medical sutures (nylon, PLGA), metal (steel) suture, self-driven electrical stimulation suture (ES-suture)[1] and BET-suture of different electrodes (Steel, Mg, Mo). The schematic illustrates the principle of evaluating bending properties.

The bending stiffness B ($cN\cdot cm^2/cm$) of the textile can be expressed as:

$$B = \frac{B_p + B_n}{2} \qquad (1)$$

The bending hysteresis moment 2HB (N cm/cm) can be expressed as:

$$2HB = \frac{2HB_p + 2HB_n}{2} \qquad (2)$$

where B_p and B_n are the average slopes of the lines with curvature K = 0.5 ~ 1.5 and -0.5 ~ -1.5, respectively; $2HB_p$ and $2HB_n$ are the bending hysteresis moments at K = 0.5 and K = -0.5, respectively.

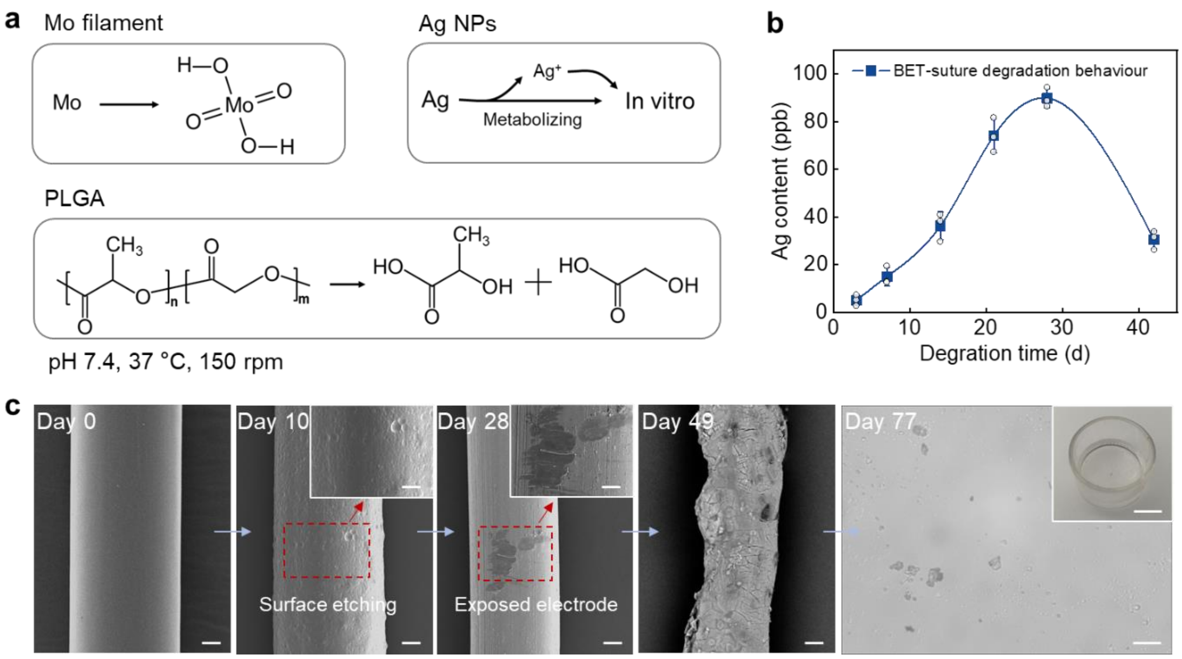

Figure S6. Degradation characteristics of BET-suture. (a) Metabolic processes of all materials composing BET-suture. Mo undergoes oxidative dissolution in physiological fluids to form soluble molybdate ions, which are subsequently excreted via renal clearance. PLGA is hydrolyzed into lactic and glycolic acids, which enter the tricarboxylic acid cycle and are metabolized to CO_2 and H_2O. In contrast, Ag NPs are not fully bioabsorbable; they undergo partial oxidative dissolution to release Ag^+, which bind to proteins or exist as free ions in plasma and are primarily eliminated through glomerular filtration and urinary excretion. (b) The relationship between the Ag content in the degradation solution and the degradation time. n = 3 independent samples. (c) Variations in the surface morphology of BET-suture during various stages of the degradation cycle. No visible BET-suture was seen in the degradation solution after 11 weeks. Scale bars: 10 µm for SEM images, 2 µm for SEM insets, 10um for microscope images, 3 cm for physical images. Data represent mean ± standard deviation.

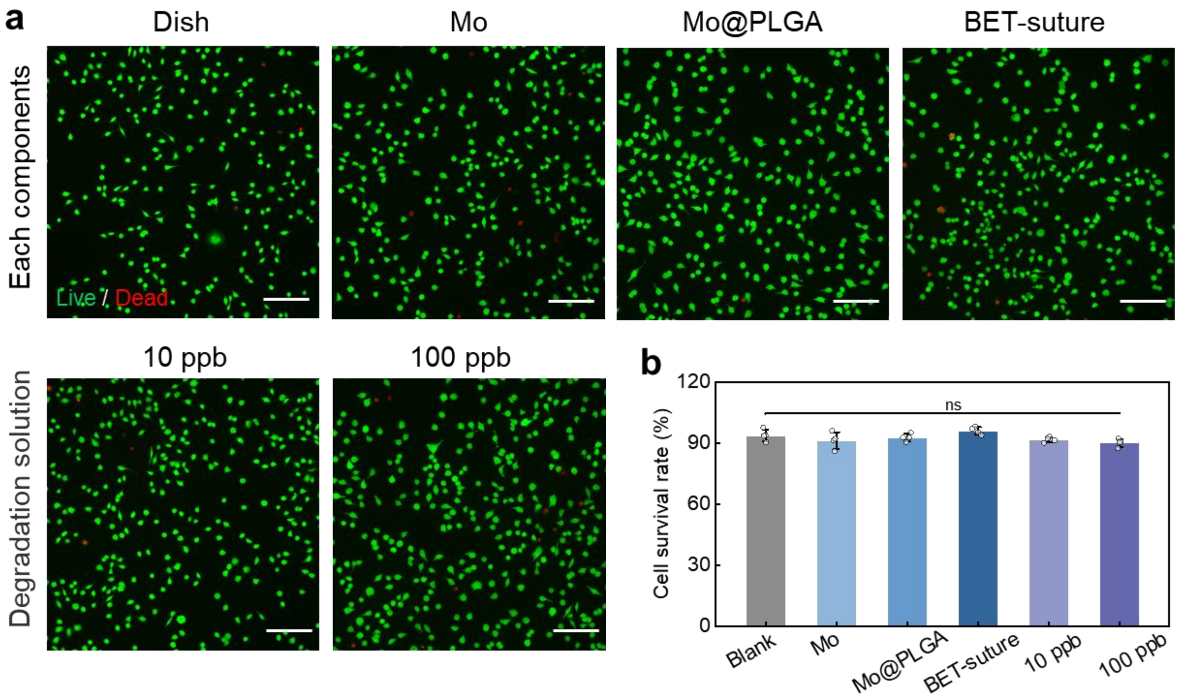

Figure S7. Biocompatibility of BET-suture and its components and degradation products. (a) Live/dead staining images of BET-suture and its components and degradation solution treating L929 cells for 3 days. 10 ppb and 100 ppb represent the different silver content in the degradation solution, respectively. Scale bar: 100 μm. (b) Proportion of live L929 cells after live/dead staining in each group. n = 4 independent samples. All statistical analyzes were performed by one-way ANOVA, data represent mean ± standard deviation, ns indicates not significant.

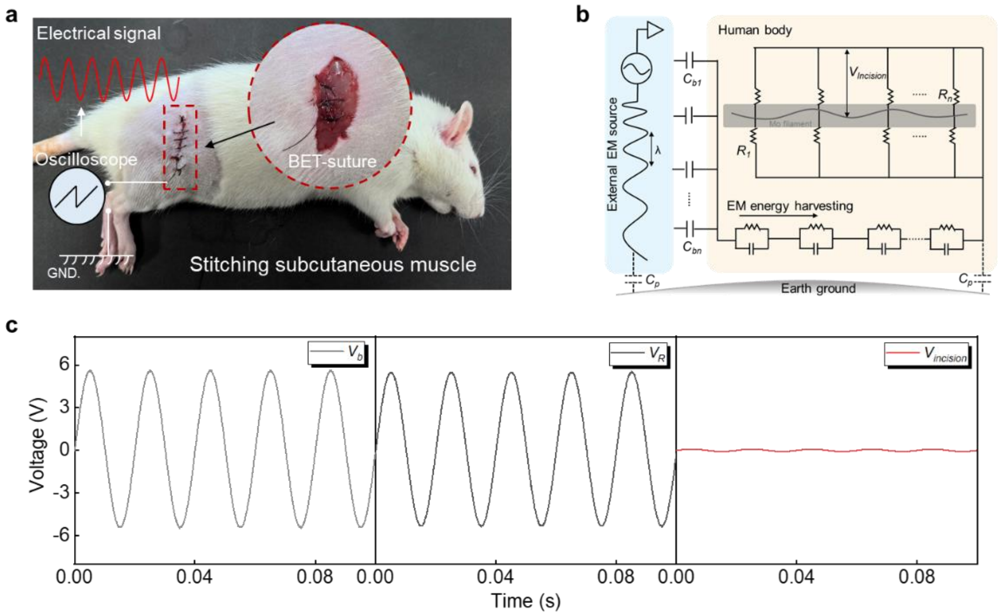

Figure S8. In vivo electrical signal testing after BET-suture implantation. (a) Optical image of BET-suture stitched to the subcutaneous muscle of SD rats (red dashed box). One end of the BET-suture was left outside the body after stitching and was used to connect to an oscilloscope for real-time measurement of electrical signals. (b) Equivalent circuit diagram of BET-suture without dielectric layer by body-coupled EM energy. C_b, body-coupled capacitance; R_n, the resistance of the muscle; C_p, parasitic capacitance between body and earth ground; $V_{incision}$, electric potential difference between body and BET-suture's core layer. (c) Real-time measurements of $V_{incision}$, V_b, and V_R during wireless electrical stimulation. V_b, open-circuit voltage of body-coupled EM energy; V_R, open-circuit voltage of BET-suture's core layer.

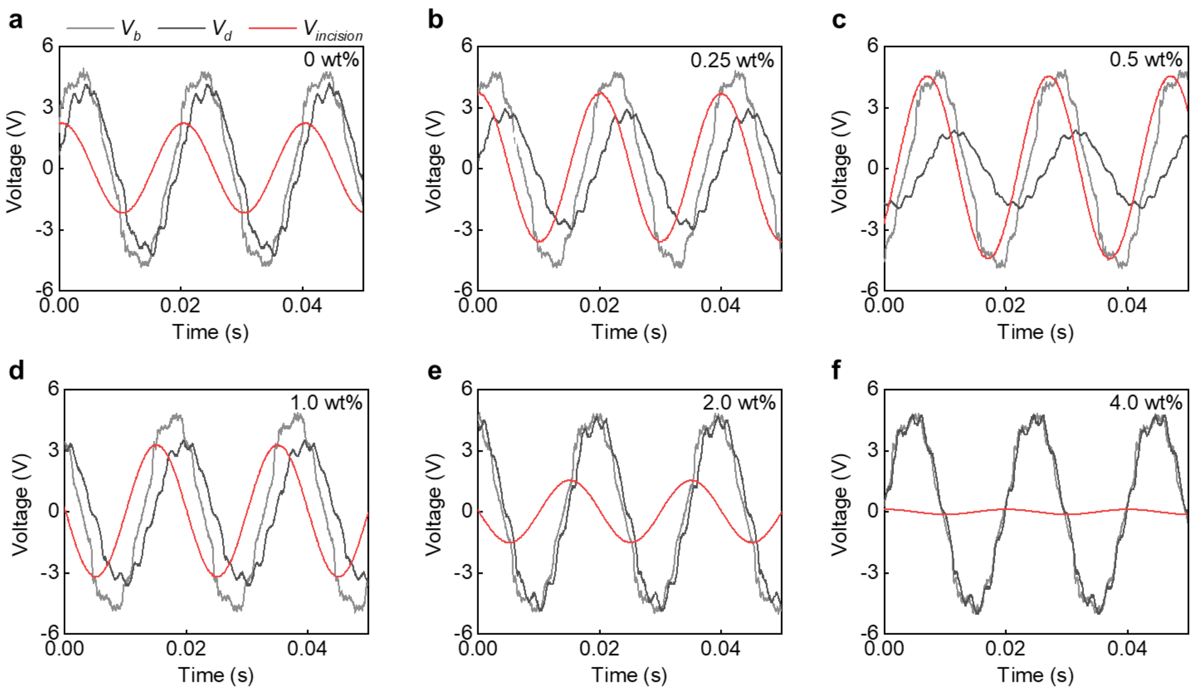

Figure S9. Effect of Ag NPs content on electrical stimulation. The V_b, V_d and $V_{incision}$ of BET-suture with Ag NPs content of (a) 0 wt%, (b) 0.25 wt%, (c) 0.5 wt%, (d) 1.0 wt%, (e) 2.0 wt% and (f) 4.0 wt% dielectric layer were measured in real time. As the content of Ag NPs increased, the $V_{incision}$ showed a tendency to increase and then decrease. The BET-suture with 0.5 wt% Ag NPs had the optimal electrical stimulation effect.

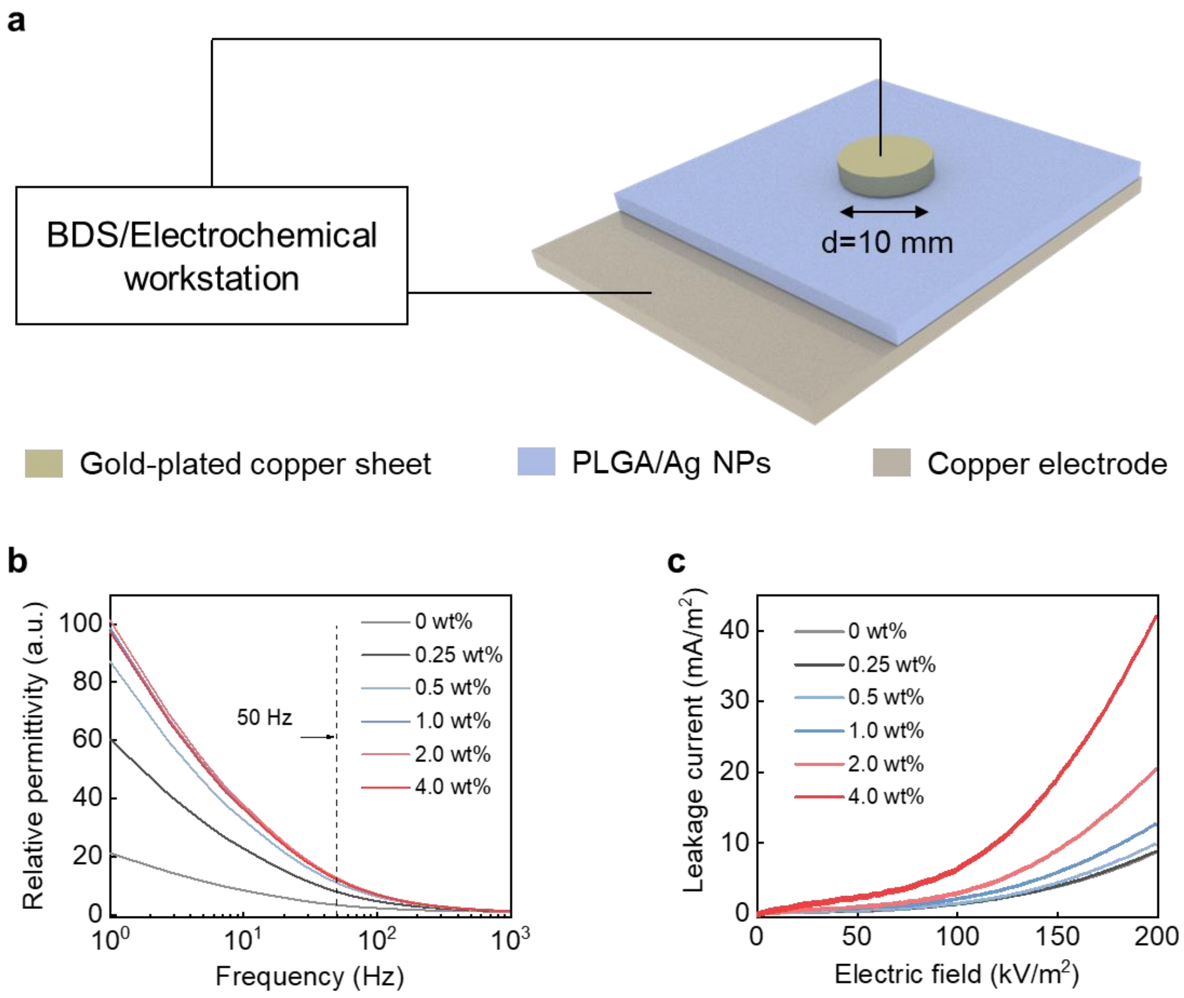

Figure S10. Dielectric properties of BET-suture's sheath layer (PLGA/Ag NPs composite). (a) Schematic diagram of the parallel-plate capacitor and experimental setup for determining the relative permittivity and leakage current density of the BET-suture's sheath layer. (b) Variation curves of relative permittivity with frequency for sheath layers with different Ag NPs contents. (c) Variation curves of leakage current density with external E-field for sheath layers with different Ag NPs contents.

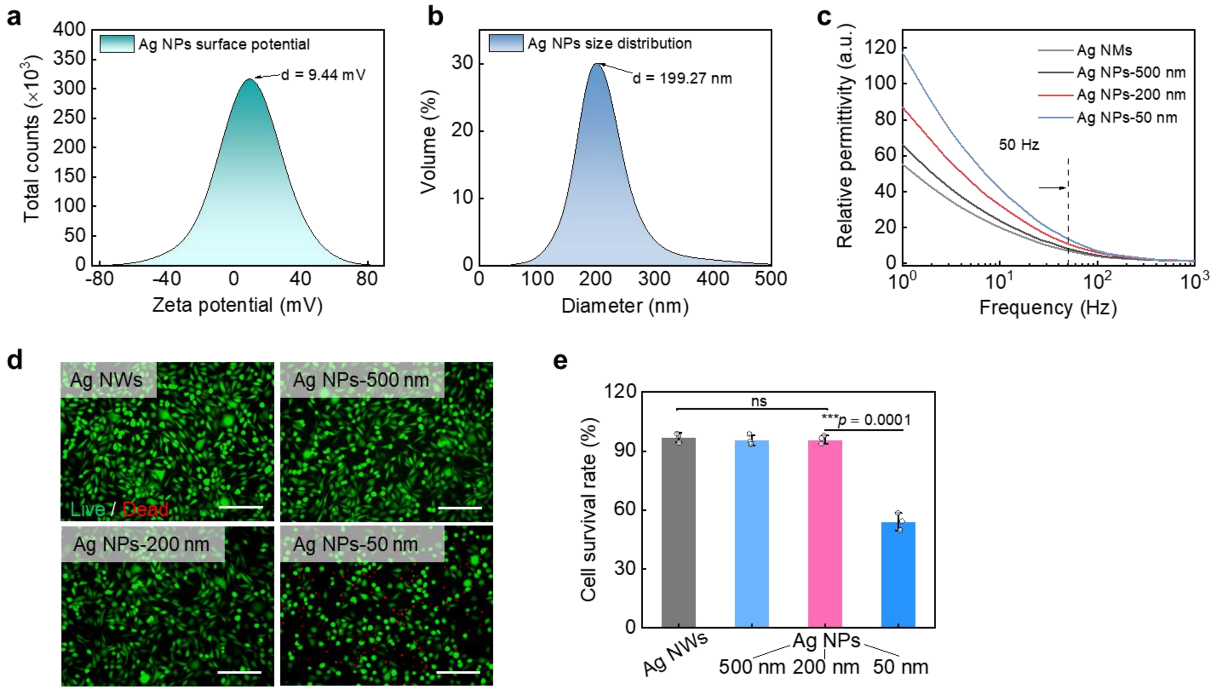

Figure S11. Relevant parameters and performance of Ag NPs and comparison of dielectric properties of different silver nanofillers. (a) Zeta potential and (b) particle size distribution of Ag NPs. (c) Relative permittivity of BET-suture sheath layers with Ag NPs of different sizes and silver nanowires (Ag NWs) as nano-doped particles. The addition amount of silver nanofillers is 0.5 wt%. (d) Live/dead staining images of Ag NPs of different sizes and Ag NWs treating L929 cells for 3 days. Dispersion concentration: 100 ppb, scale bar: 100 μm. (e) Proportion of live L929 cells after live/dead staining in each group. n = 3 independent samples. All statistical analyzes were performed by one-way ANOVA, data represent mean ± standard deviation, ***p < 0.001, ns indicates not significant.

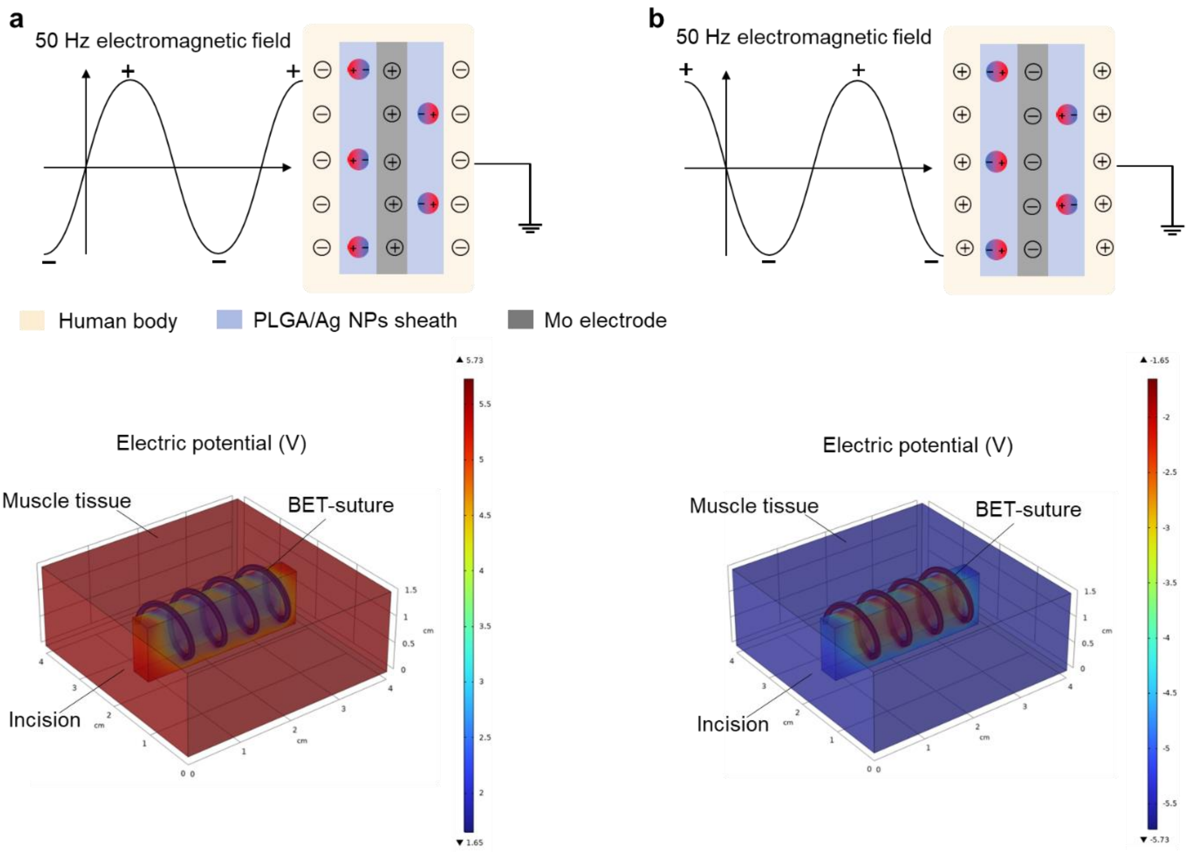

Figure S12. COMSOL simulation of the E-field formed at the incision during different periods of BET-suture. (a) Positive potential E-field. (b) Negative potential E-field. The simulation results showed that BET-suture could generate persistent alternating changing E-fields at a set frequency and act on the wound tissue.

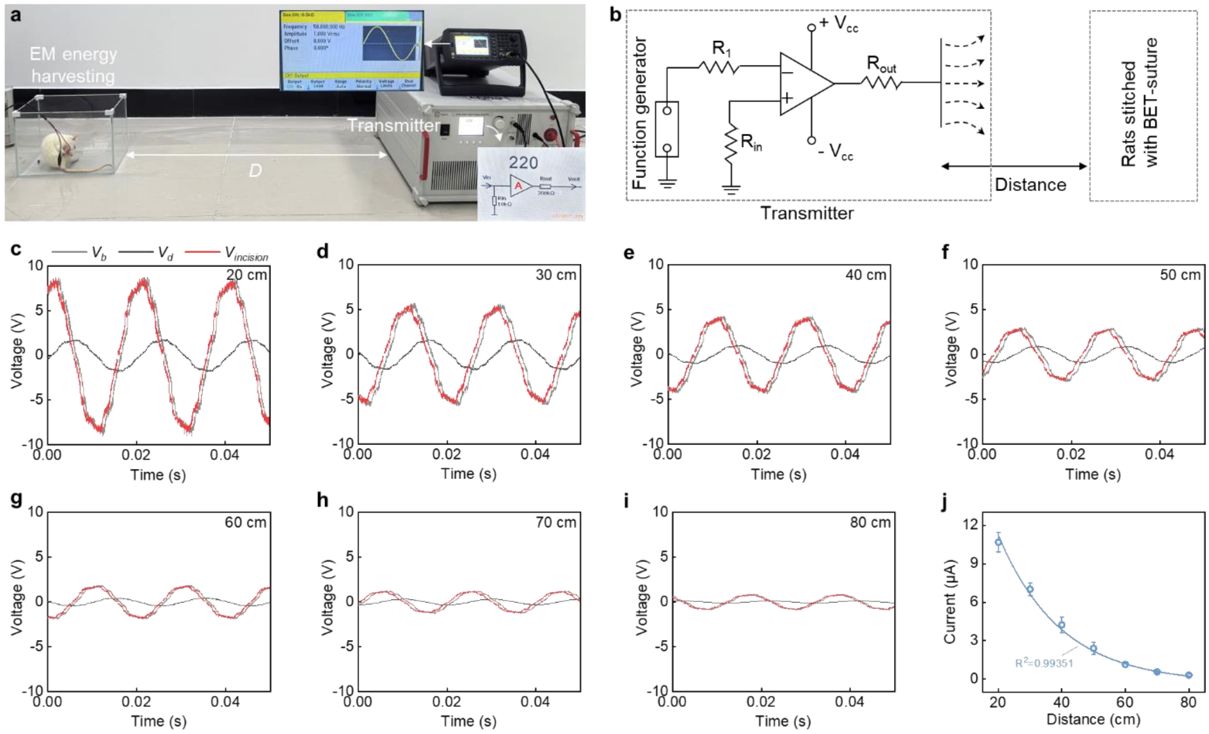

Figure S13. Effect of distance of BET-suture from electromagnetic field on electrical properties. (a) Physical diagram of the experimental setup for measuring electrical signals. The transmitter was used to simulate an electromagnetic field in the environment (23.8 dBm, 50 Hz). D represents the distance between the BET-suture-stitched SD rat and the transmitter. (b) Schematic diagram of the generation and flow of wireless signals. V_b, V_d and $V_{incision}$ were measured at distances of (b) 20 cm, (c) 30 cm, (d) 40 cm, (e) 50 cm, (f) 60 cm, (g) 70 cm and (h) 80 cm from the transmitter. (j) Peak current (I_p) of ES current ($I_{incision}$) at different distances from the transmitter. n = 3 independent samples. Data represent mean ± standard deviation.

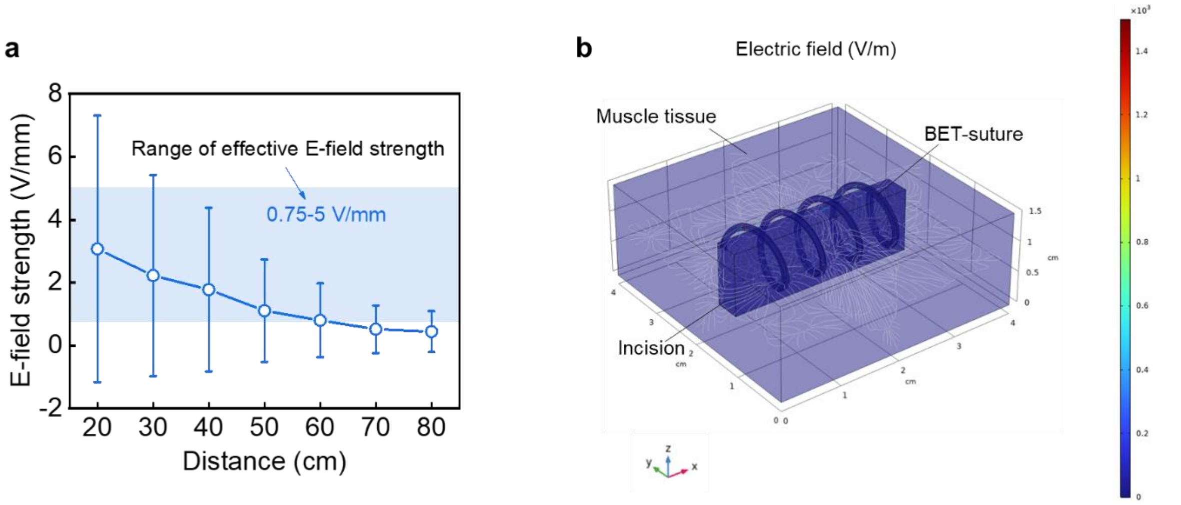

Figure S14. COMSOL simulation of the E-field of BET-suture without dielectric layer at the incision and average electric field strength at different distance. (a) The maximum, minimum and median values were taken from the E-field results of the COMSOL simulation and the average E-field strength was calculated. (b) Distribution of E-field when BET-suture without dielectric layer stimulates the incision. Obviously, the removal of the dielectric layer makes the potential between the suture and the tissue consistent and does not create a potential difference. Therefore, BET-suture would not generate a pro-healing endogenous E-field at the wound. Data represent mean ± standard deviation.

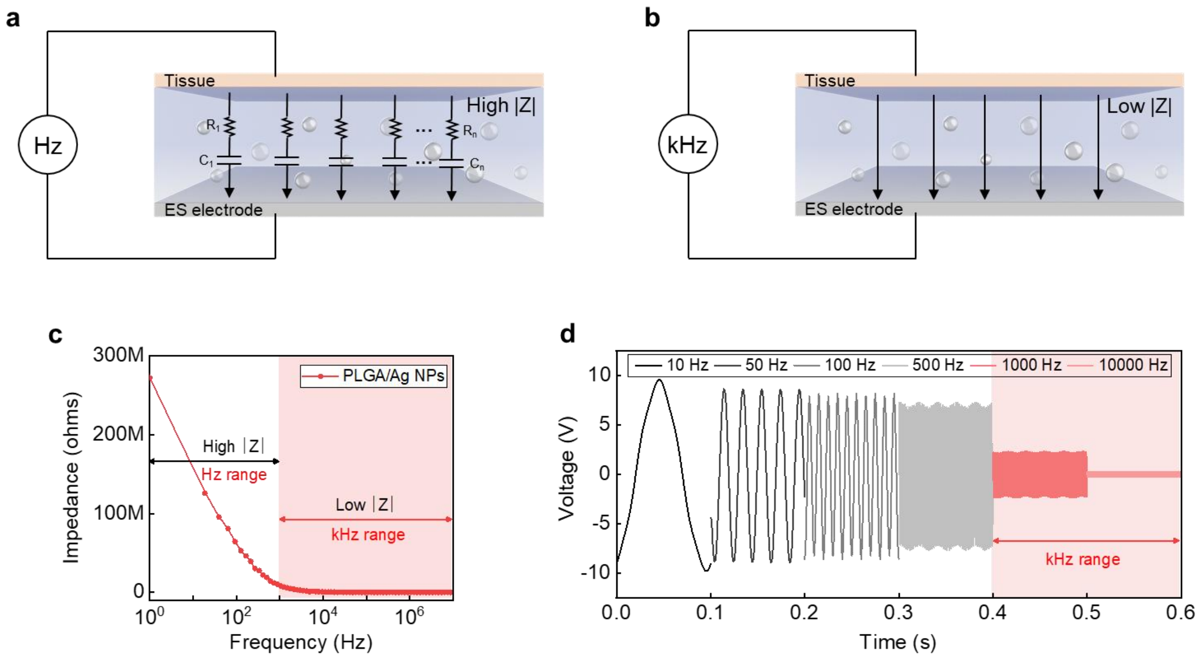

Figure S15. Effect of frequency on the electrical properties of BET-suture. Equivalent circuits of the dielectric layer at (a) Hz and (b) kHz frequencies. High impedance (|Z|) and stored charge at low frequency; low |Z| and charge loss at high frequency. (c) Frequency impedance spectrum of BET-suture's dielectric layer. (d) Real-time measurement of $V_{incision}$ at different frequencies.

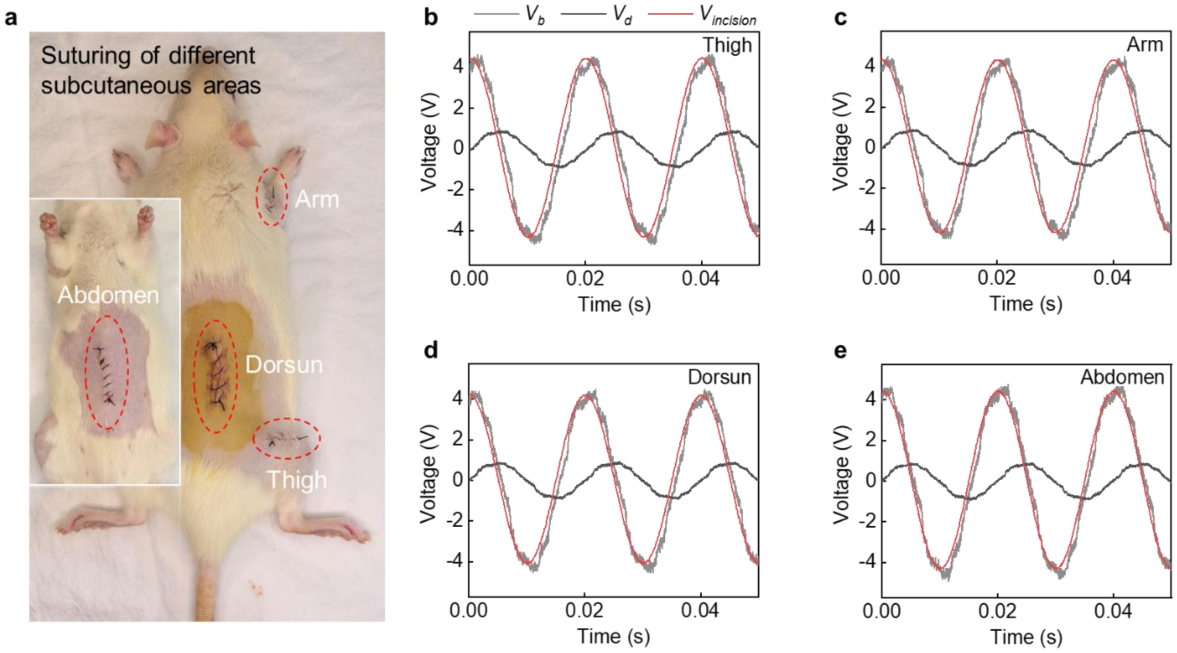

Figure S16. Electrical properties of BET-suture at different stitching sites. (a) Physical image of BET-suture for subcutaneous stitching of different sites in SD rats. Real-time measurement of V_b, V_d and $V_{incision}$ after BET-suture stitching sites, including (b) thigh, (c) arm, (d) dorsun, (e) abdomen. The $V_{incision}$ of the four sites was similar, indicating that the BET-suture is suitable for a wide range of surgical scenarios.

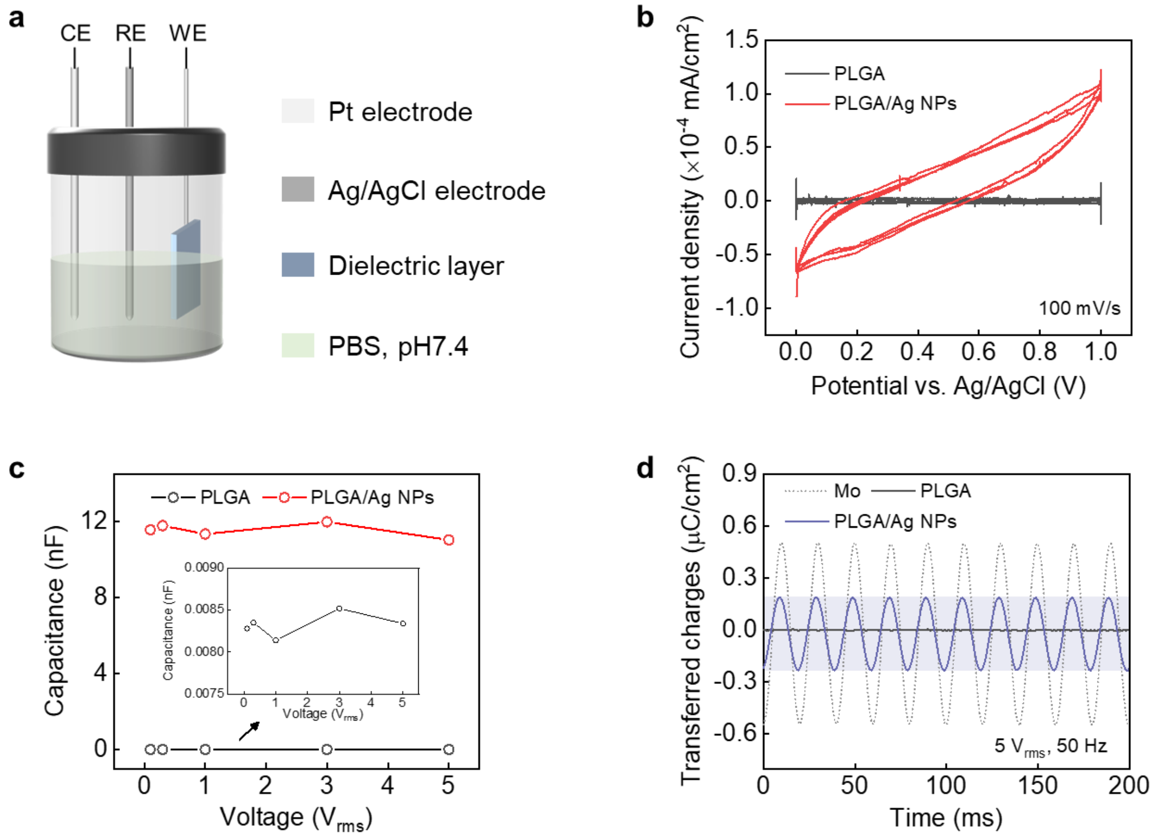

Figure S17. Capacitive properties of BET-suture's dielectric layer. (a) Measurement of electrochemical properties of the dielectric layer using a three-electrode electrochemical cell. (b) CV cycling curves and (c) capacitance of the dielectric layers containing Ag NPs and without Ag NPs. (d) Real-time transferred charge densities of Mo, PLGA and PLGA/Ag NPs at a fixed AC. Mo serves as the control reference (gray dashed line) to quantify the transient charge transfer density from the signal generator.

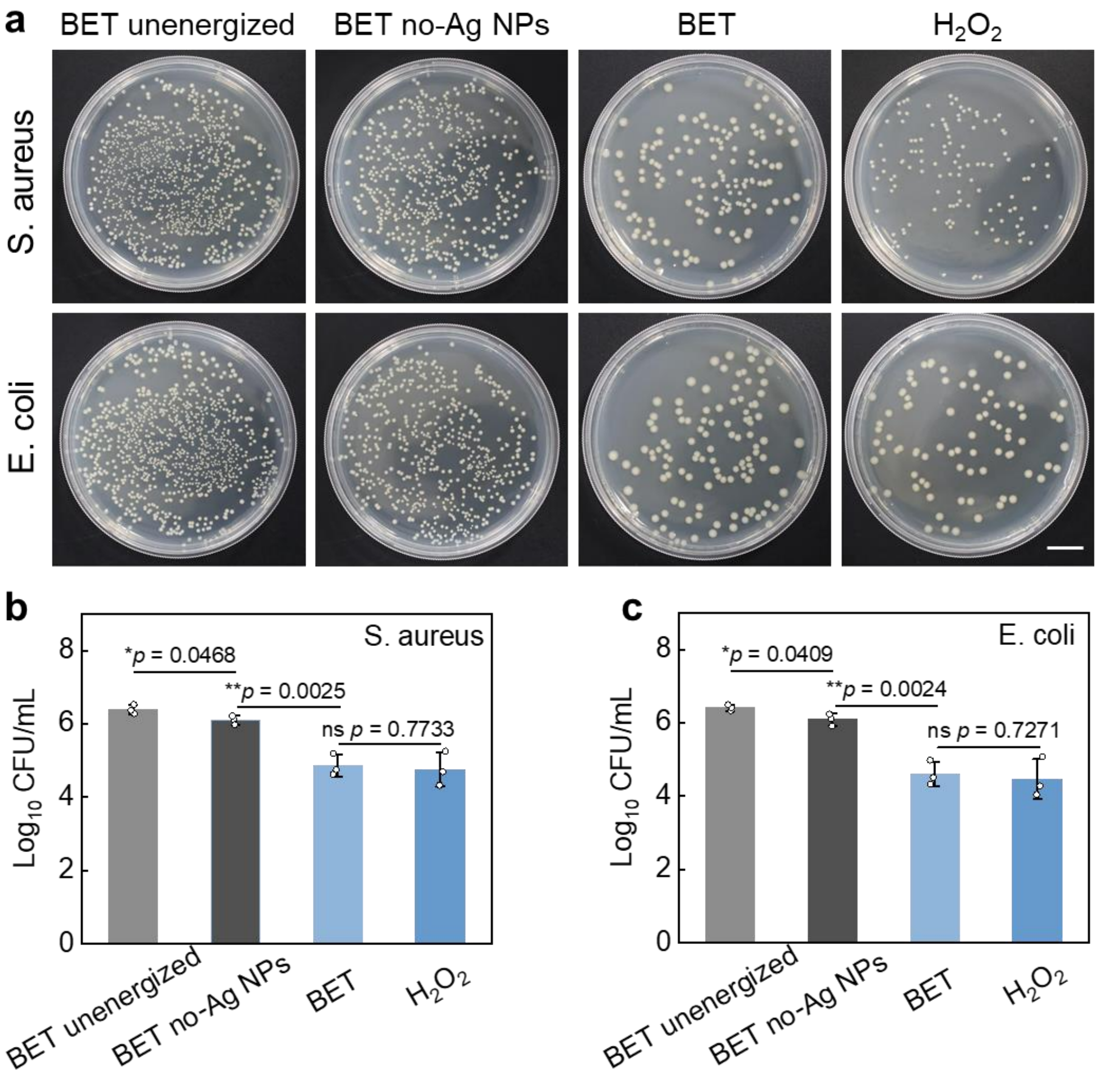

Figure S18. Capacitive antimicrobial capacity of BET-suture. (a) Photographs of the proliferative state of S. aureus and E. coli on LB plates after treatment in each group. Scale bar: 1 cm. cell counting statistics for (b) S. aureus and (c) E. coli on LB plates. n = 3 independent samples. All statistical analyzes were performed by one-way ANOVA, data represent mean ± standard deviation, $**p < 0.01$, $*p < 0.05$, ns indicates not significant.

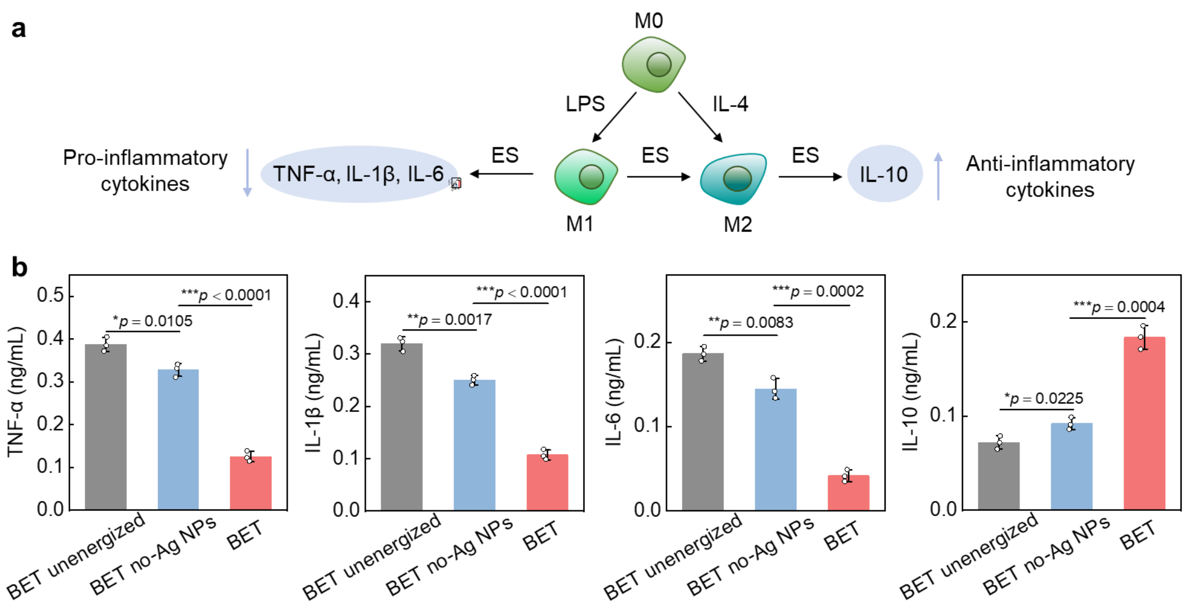

Figure S19. Anti-inflammatory effects of capacitive ES of BET-suture. (a) Anti-inflammatory mechanism of BET-suture. Capacitive ES further promoted the secretion of anti-inflammatory factors, inhibited the action of pro-inflammatory factors, and stimulated the conversion of macrophages from M1 type to M2 type. (b) Cytokine expression levels in macrophages under different treatment conditions. n = 3 independent samples. All statistical analyzes were performed by one-way ANOVA, data represent mean ± standard deviation, ***p < 0.001, **p < 0.01, *p < 0.05.

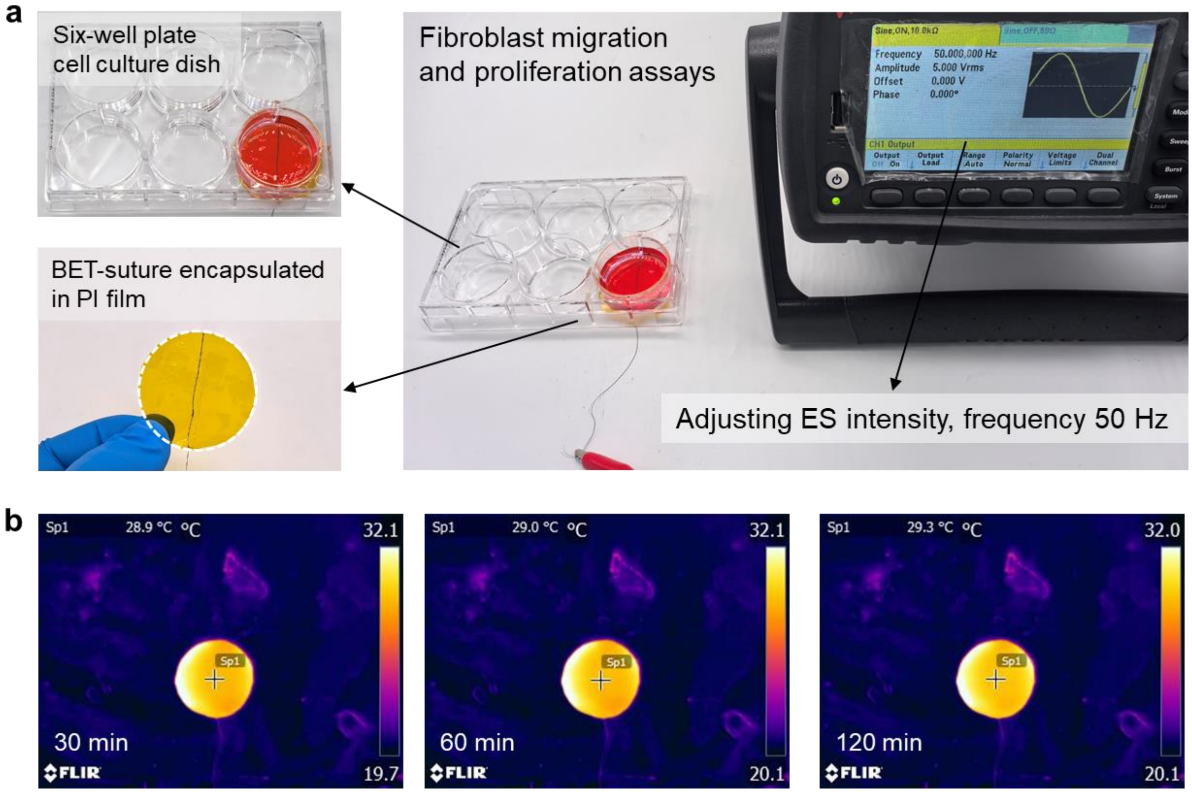

Figure S20. Fibroblast proliferation and migration test under electrical stimulation by BET-suture. (a) A RF/microwave signal generator was used to simulate the power supply of electrical stimulation and connected to the electrodes of BET-suture. BET-suture was encapsulated with PI films and placed on the bottom of six-well plates, and the ES intensity was adjusted for the test. (b) Infrared thermography photos of the BET-suture encapsulated PI films in continuous operation for 2 hours.

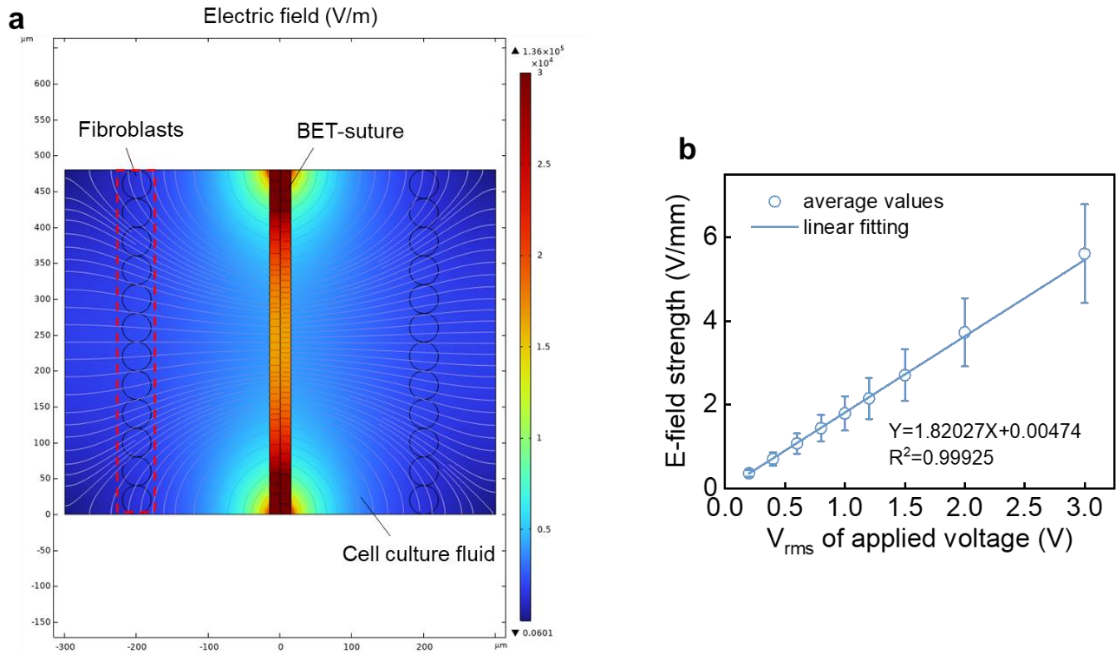

Figure S21. COMSOL simulation of the E-field distribution of BET-suture in cell petri dish. (a) Simulation of the E-field strength of cells at a distance of 200 μm from the BET-suture after energization. (b) The average E-field strength experienced by fibroblasts was calculated from the simulation results and the function was fitted to guide the execution of the cellular experiments.

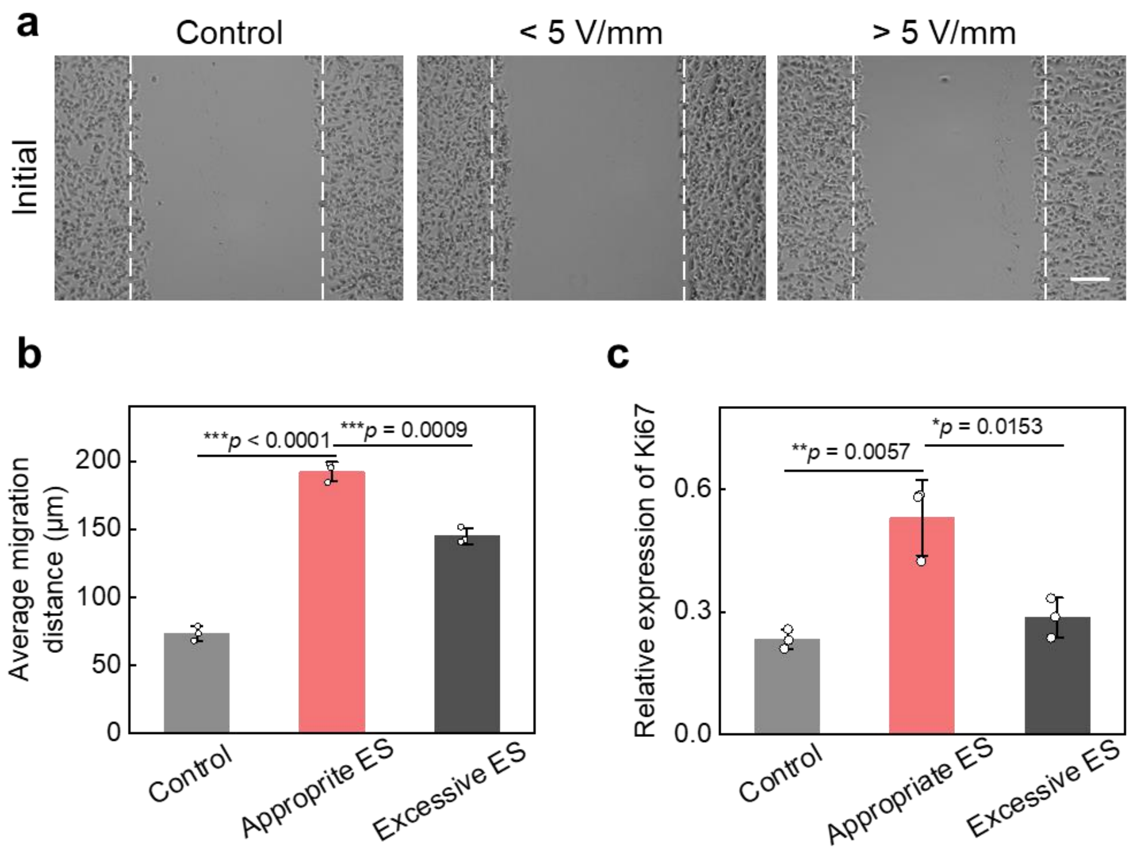

Figure 22. Proliferation and migration of fibroblasts under ES. (a) Initial microscopic images of the scratch experiment for each group. Scar bar: 80 μm. (b) Statistics of the average migration distance of fibroblasts in each group after 24 h. (c) Relative expression of Ki67-stained cells in each group after 72 hours. n = 3 independent samples. All statistical analyzes were performed by one-way ANOVA, data represent mean ± standard deviation, ***p < 0.001, **p < 0.01, *p < 0.05.

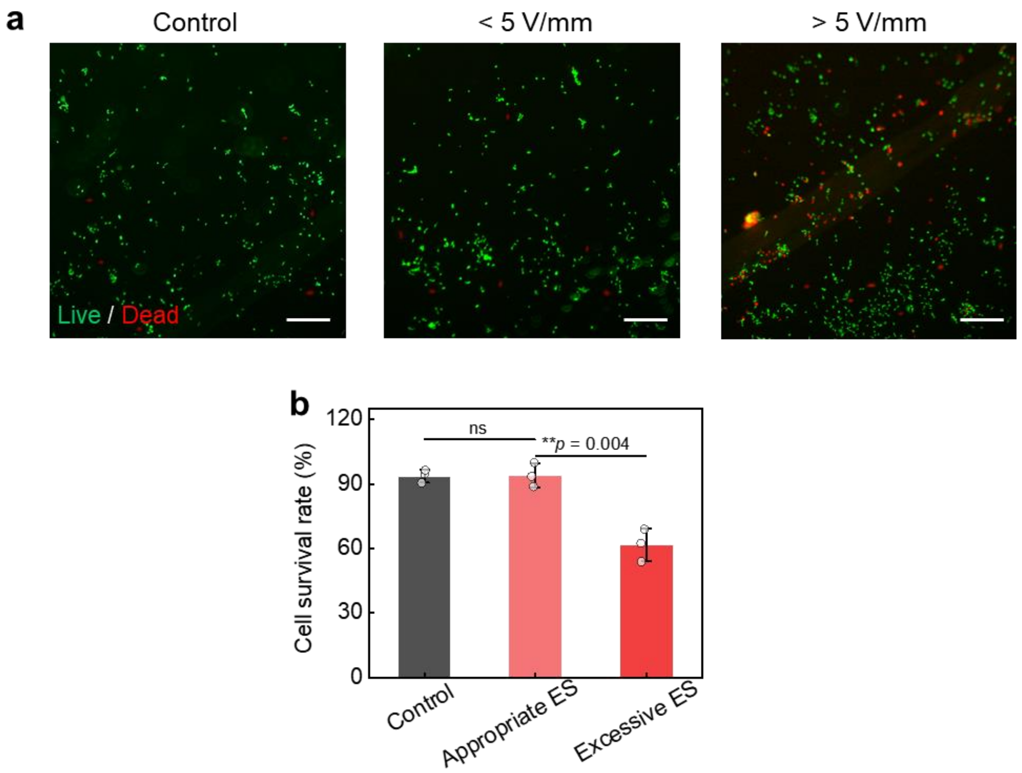

Figure S23. Effect of E-field strength on cell proliferation provided by BET-suture. (a) Live/dead fluorescence staining of cells stimulated with different E-field strengths after 72 hours. Scale bar: 500 μm. (b) Cell survival statistics of fluorescent staining results. All statistical analyzes were performed by one-way ANOVA, data represent mean ± standard deviation, **p < 0.01, ns indicates not significant.

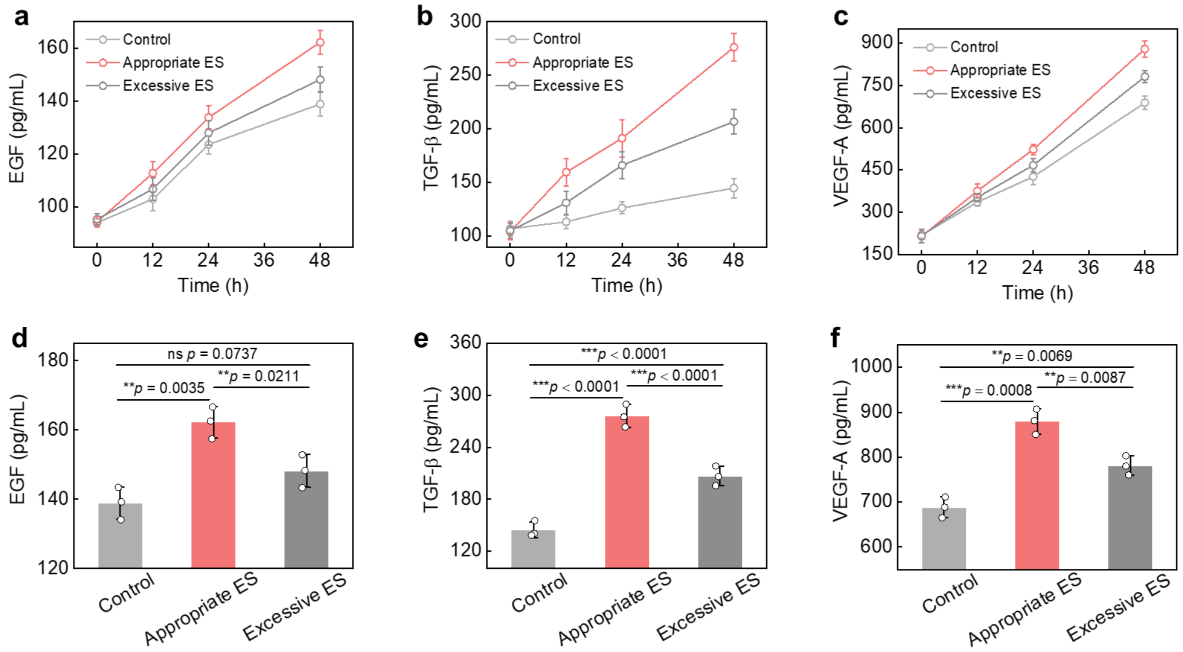

Figure S24. Effect of electrical stimulation on cell growth factor secretion. ELISA assay for quantifying the contents of (a) EGF, (b) TGF-β and (c) VEGF-A in each group at different times. Comparison of the contents of (d) EGF, (e) TGF-β and (f) VEGF-A among the groups after 48 h of ES action. n = 3 independent samples. All statistical analyzes were performed by one-way ANOVA, data represent mean ± standard deviation, ***$p < 0.001$, **$p < 0.01$, *$p < 0.05$, ns indicates not significant.

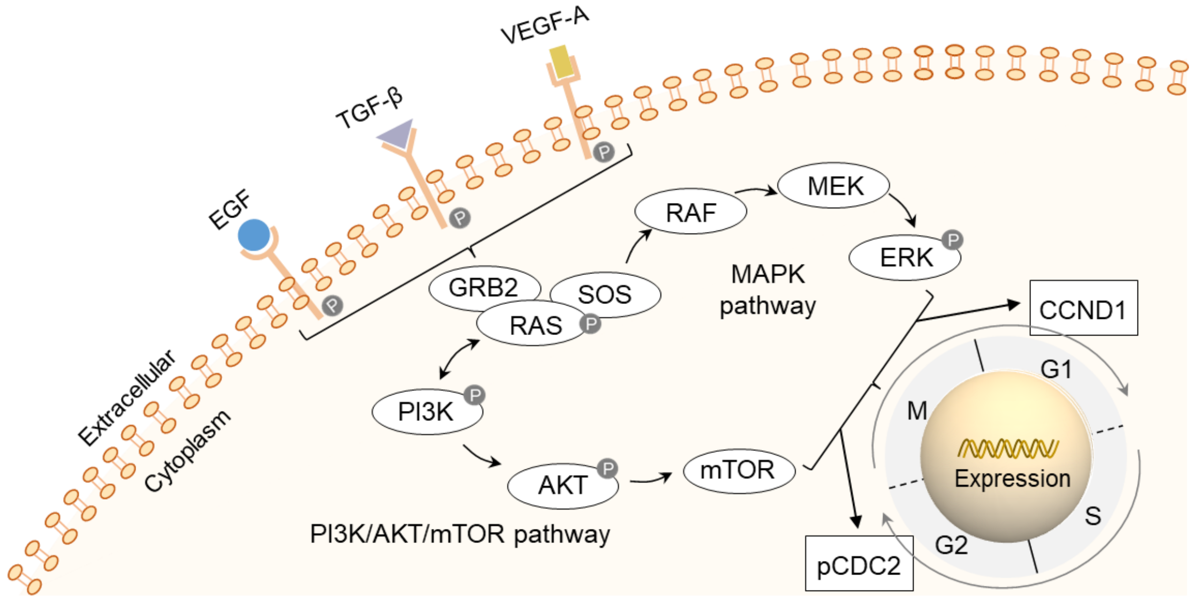

Figure S25. Effective ES of BET-suture regulated the acting mechanism of the proliferative phase. ES activates exocytosis behaviour, induces the production of specific signals (e.g. Ca^{2+} signals), enhances the secretion and expression of relevant growth factors (EGF, TGF-β, VEGF-A), activates signaling pathways (PI3K/AKT/mTOR and MAPK signaling pathways) regulating cell proliferation and promotes the expression of cell-cycle proteins, thus enhancing the process of cell proliferation and increasing the efficiency of healing.

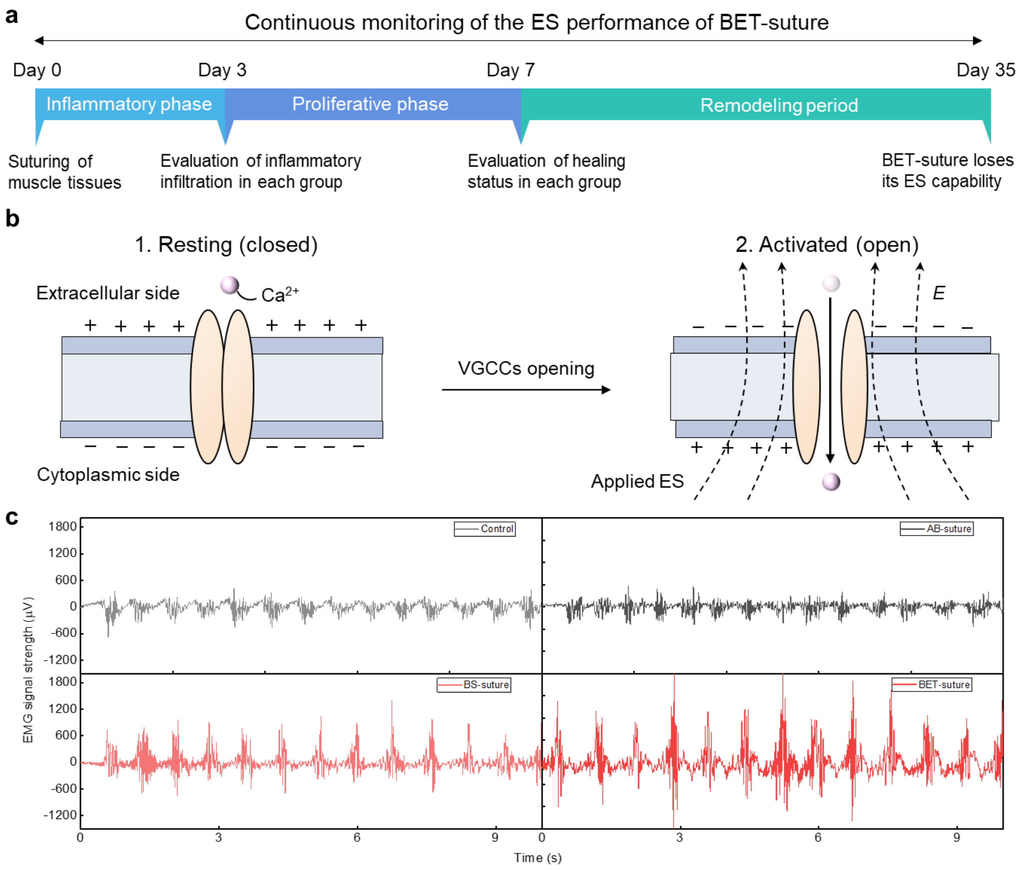

Figure S26. Changes in electromyographic (EMG) signals under the effect of electrical stimulation by BET-suture. (a) The schematic timeline of the animal experiment protocol. (b) Mechanism of the effect of applied ES on voltage-guided calcium channels (VGCCs). ES alters the cell membrane potential, which in turn activates VGCCs, triggering the inward flow of calcium ions, forming intracellular calcium signals and enhancing the strength of EMG signals. (c) Real-time EMG signals of different suture groups.

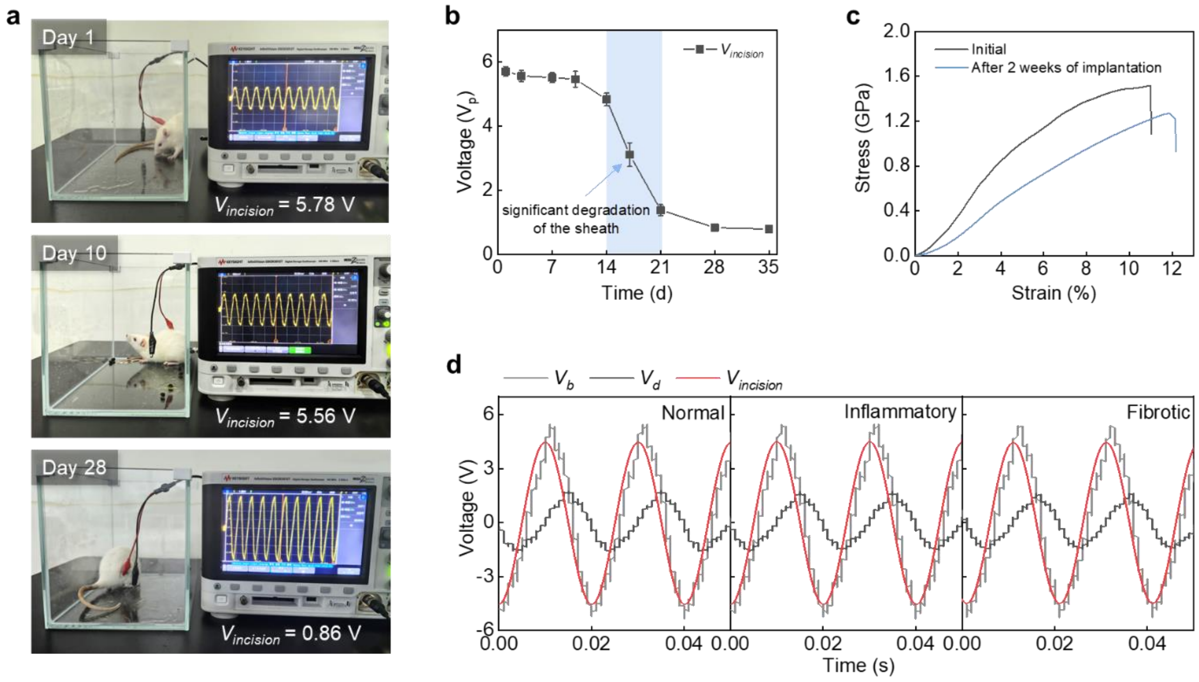

Figure S27. Electrical stability of BET-suture in vivo after implantation. (a) Photos of $V_{incision}$ measured in real time after 1, 10 and 28 days of BET-suture implantation. (b) Variation of V_p at different times of implantation. The results showed that the BET-suture could provide stable electrical stimulation and stitching force during the muscle recovery period (10 days). After 4 weeks, the dielectric layer of BET-suture was degraded and the electrical stimulation performance was lost. n = 3 independent samples. (c) Stress-strain curves of BET-suture before and after 2 weeks of implantation. (d) Real-time measurements of $V_{incision}$, V_b, and V_d in different states of muscle tissue during wireless ES. Data represent mean ± standard deviation.

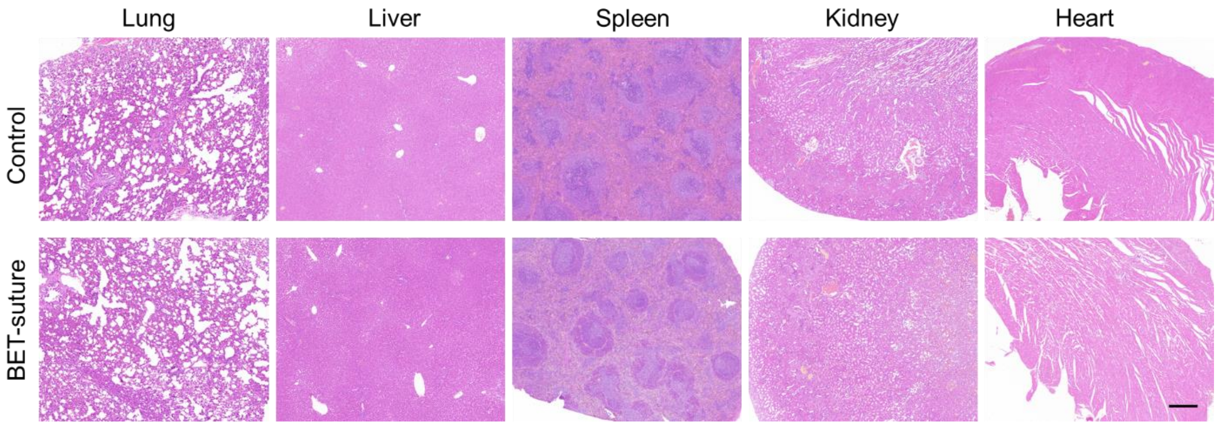

Figure S28. H&E staining of vital organs (lung, liver, spleen, kidneys and heart) in normal rats and BET-suture rats after 28 days of stitching. This indicated that the low amount of Ag NPs added in the BET-suture exhibited good biocompatibility and low toxicity. This addressed the biological hazards associated with excessive addition of Ag NPs as antibacterial agents in previous studies, providing experimental evidence for the safe design of silver-based nanocomposites. Scar bar: 300 μm.

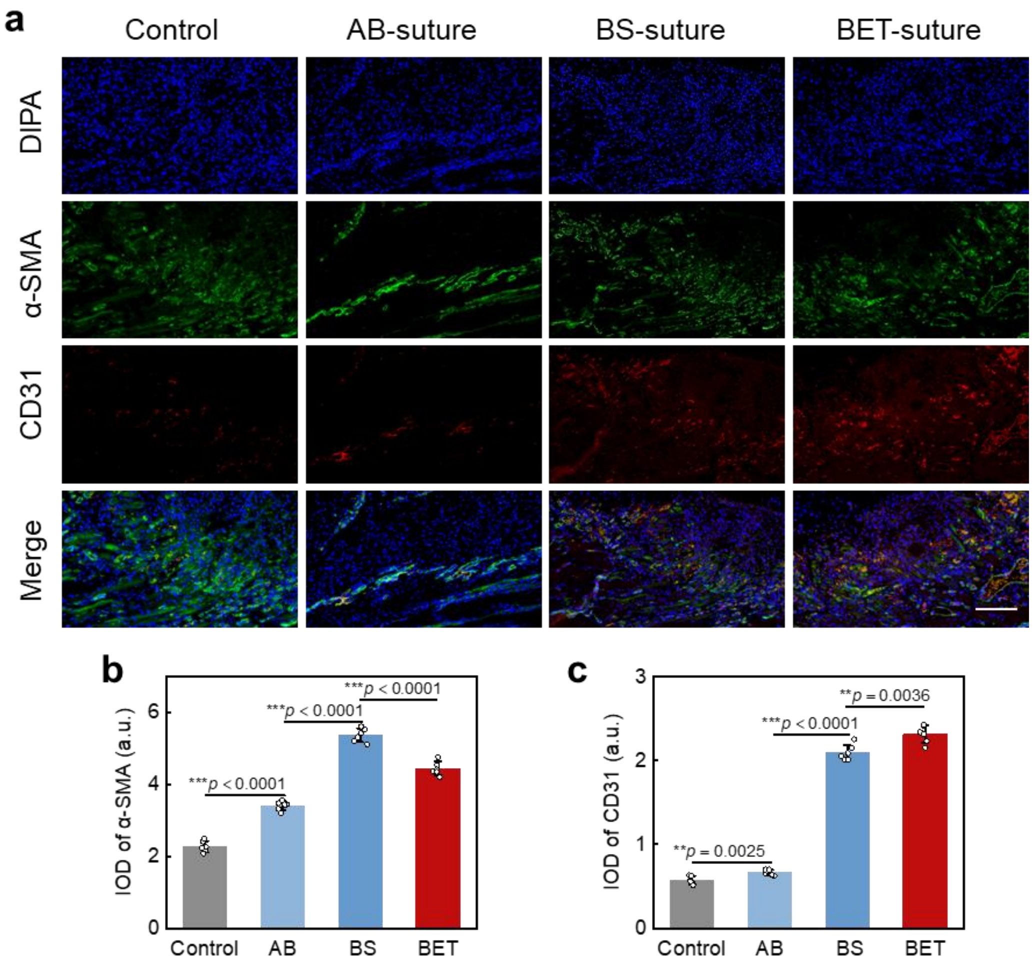

Figure S29. Evaluation of cellular status of healing tissues and angiogenic density in vivo. (a) Immunofluorescence staining images of α-SMA and CD31 in each group. Quantitative statistics of (b) α-SMA and (c) CD31 immunofluorescence staining results in each group. Scar bar: 200 μm. n = 6 independent samples. All statistical analyzes were performed by one-way ANOVA, data represent mean ± standard deviation, $^{***}p < 0.001$, $^{**}p < 0.01$.

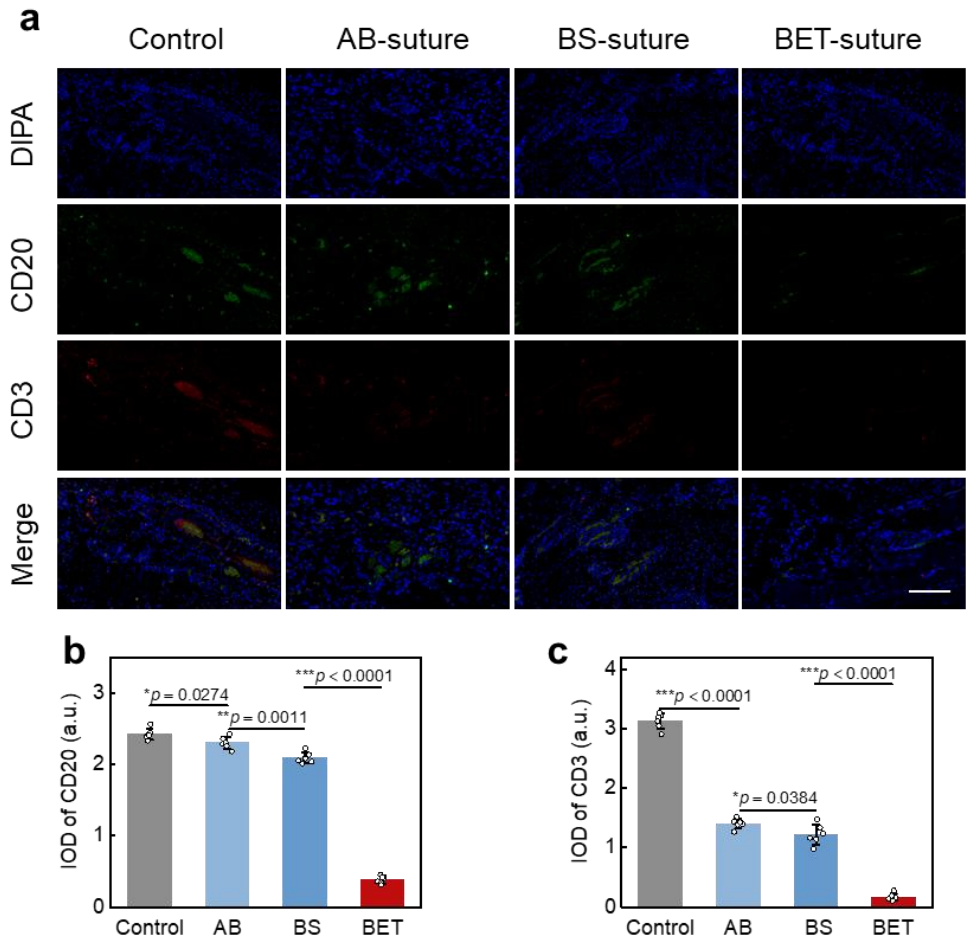

Figure S30. Evaluation of inflammatory infiltration in healing tissues. (a) Immunofluorescence staining images of CD20 and CD3 in each group. Quantitative statistics of (b) CD20 and (c) CD3 immunofluorescence staining results in each group. Scar bar: 200 μm. n = 6 independent samples. All statistical analyzes were performed by one-way ANOVA, data represent mean ± standard deviation, ***p < 0.001, **p < 0.01, *p < 0.05.

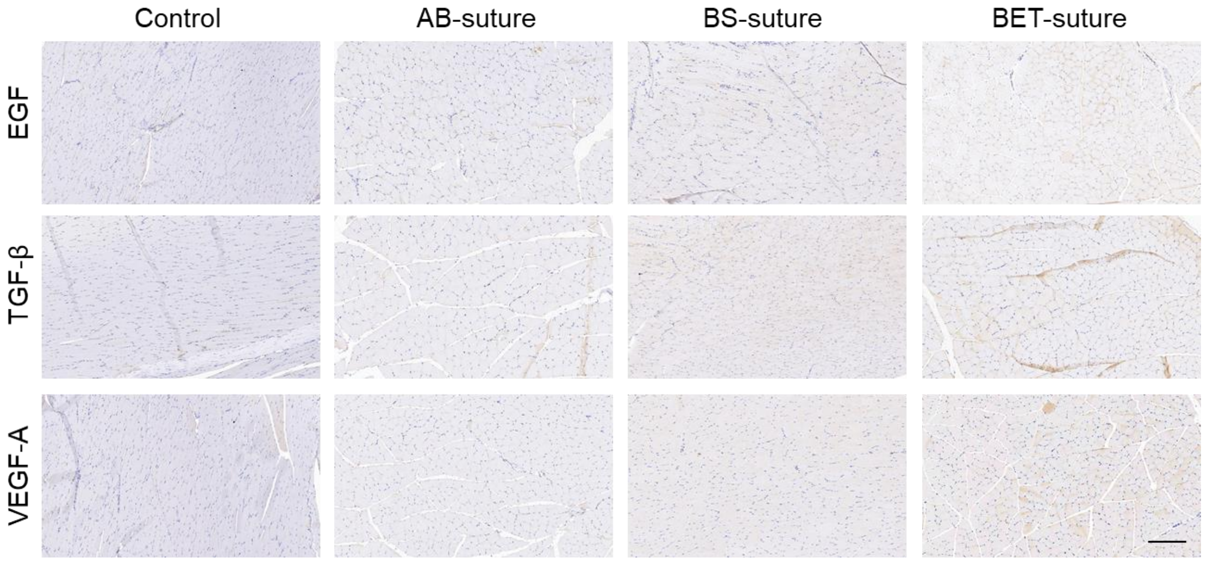

Figure S31. Immunohistochemistry staining of each group. Representative immunohistochemical images of healing tissue for EGF, TGF-b, and VEGF-A growth factors. Scar bar: 200 μm.

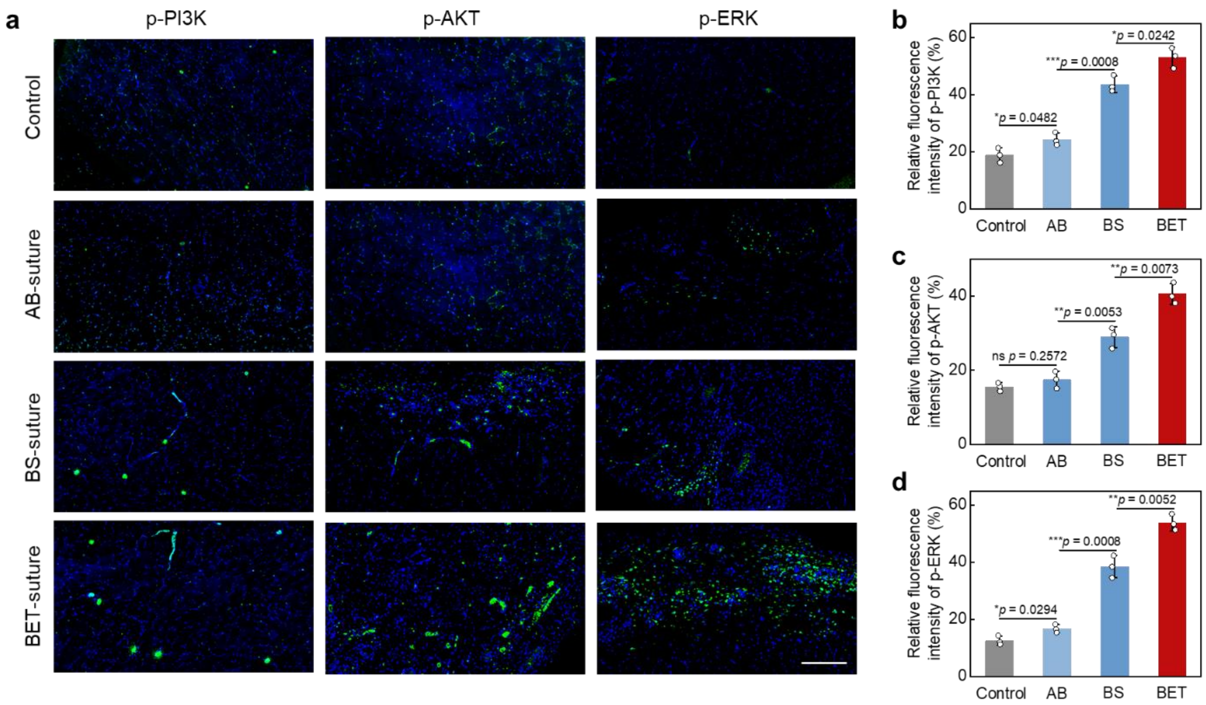

Figure S32. Expression analysis of related proteins in signaling pathways. (a) Fluorescence staining images of p-PI3K, p-AKT, p-ERK in the healing tissues of rats in each group. Relative expression of phosphorylated proteins in the fluorescent staining results of each group, including (b) p-PI3K, (c) p-AKT, (d) p-ERK. Scar bar: 200 μm. n = 3 independent samples. All statistical analyzes were performed by one-way ANOVA, data represent mean ± standard deviation, ***p < 0.001, **p < 0.01, *p < 0.05, ns indicates not significant.

Supplementary Tables

Table S1. Mechanical properties of state-of-the-art high-strength and functionalized sutures.

Suture type	Function	In vivo absorption	Elongation (%)	Stiffness (N/mm)	Ref.
PET	Ligament stitching	No	41-53	63.98	[2]
Carbon			11-17	224.76	
Autograft			13.0-86.1	17-106.5	
HHF			0.472	79.43	
HyDe-gut	Pharmaceutical loading	Yes	10.0-32.5	4.5-16.6	[3]
PGLAS-NFs-K18	Photodynamic therapy				[4]
ES-suture	Mechanoelectric ES		10.06	25.04	[1]
**BET-suture**	**Wireless ES**		**11.37**	**21.7-63.9**	**This work**

Table S2. Driving means and principles of various types of electrical stimulation.

Source of ES	Device shape and materials	ES power consumption	ES formation process	Ref.
External power supply	Patch: Mg, Mo, PLGA, PA	>10^2 mW	The electrodes are connected to an external power source to create an E-field	[5]
	Patch: MoS_2, Al_2O_3, HfO_2			[6]
Primary cell	Bandage: Mo, Mg, PCL, chitosan	10 μW-10^2 mW	Asymmetric electrodes encapsulated in a gel form a primary battery applying an E-field	[7]
	Patch: PVA/PBS, Mg, PAM-MXene-SF			[8]
	Patch: ecoflex, SMA grids, PTFE electret		Oppositely charged electret forms an E-field	[9]
NFC-driven	Bandage: flexible circuit boards	1 μW-2 mW	Using NFC to power electrodes on a circuit board to create an E-field	[10]
Ultrasound-driven	Patch: PHBV/PEG, Mg	10 μW-10 mW	Ultrasound-driven mechanoelectric conversion to form an E-field	[11]
	Patch: PCL-r-PU, PEDOT:PSS, PAV, Mo			[12]
Force-driven	Patch: Mg, PLGA	10 nW-10^2 μW	Force triggers piezoelectric or triboelectric effects to produce an E-field	[13]
	Patch: collagen, PLLA			[14]
	**Suture**: Mg, PLGA, PCL			[1]
**Self-driven wireless**	**Suture: Mo, PLGA, Ag NPs**	**10 nW-10^2 μW**	**Body-coupled ambient electromagnetic energy drives suture to generate an E-field**	**This work**

Table S3. State-of-the-art therapeutic sutures and their properties and mechanisms of action.

Suture materials	Diameter (mm)	Therapeutic techniques	Repair-related signaling pathways	Promote recovery	Bioabsorbable	Ref.
PVA, Ppy, SNAP	0.1-0.4	Drugs or/and growth factors release	Inflammatory cytokines	Reduce infection, without significantly shortening the healing cycle	No	[15]
Silk, GQDs, GM-CSF, CNT	0.22				No	[16]
PGLA, CS, alginate-PAAm	0.54				No	[17]
Tissue fiber, Alg-NHS	0.39				Yes	[3]
PAMPS-AR$_x$	0.22	Enhanced biocompatibility			No	[18]
PGLAS-NFs-K18	0.38	Photodynamic therapy			No	[4]
Mg, PLGA, PCL	0.35	Passive ES	PI3K/Akt/mTOR MAPK	Healing speed increased by 1.58 times	Yes	[1]
**Mo, PLGA, Ag NPs**	**0.05-0.5**	**Capacitive antibacterial and controllable ES**	**Cellular electrophysiology PI3K/Akt/mTOR MAPK Inflammatory cytokines**	**Healing speed increased by 1.92 times with no obvious infection**	**Yes**	**This work**

Table S4. Antibody information for WB experiments.

Antibody Name	Brand	Catalog number	Molecular weight	Primary antibody dilution ratio	Secondary antibody dilution ratio
p-PI3K	Affinity	AF3241	80,84KDa	1:1000	1:5000
PI3K	Proteintech	20584-1-Ap	110-130KDa	1:1000	1:5000
p-AKT	Affinity	AF0016	60KDa	1:1000	1:5000
AKT	huabio	ET1609-51	60KDa	1:2000	1:5000
p-ERK	Affinity	AF1015	42,44KDa	1:1000	1:5000
ERK	Affinity	BF8004	42,44KDa	1:1000	1:5000
CCND1	Affinity	DF6386	34KDa	1:1000	1:5000
p-CDC2	Affinity	AF3108	30,34KDa	1:1000	1:5000
β-actin	Proteintech	66009-1-Ig	42KDa	1:2000	1:5000

Supplementary Notes

Note S1. BET-suture realizes body-coupled systems for implantable applications

There are several studies that utilize body-coupled system for various applications. Li et al.[19] designed an EM energy harvesting circuit and transmitted electricity through body coupling to power wearable electronic devices such as watches. Subsequently, Yong et al.[20] used copper patches that couple EM energy to stimulate muscles and improve muscle fatigue. Similarly, Kang et al.[21] employed a similar patch device to couple EM energy for skin wound healing. Most recently, our research group[22] designed a fiber that can utilize EM energy for human-environment interaction, achieving a chipless interaction mode.

However, all such energy interaction modes were realized through external media between the human body and the environment, without delving into the interaction form and application value of implantable electronic devices and electromagnetic energy.

In this work, the key novelty lies in pioneering a bioabsorbable, implantable fiber-based system, which for the first time proposes the in-vivo conversion mechanism of body-coupled EM energy. Specifically, it utilizes the energy storage properties of sheath layer with high dielectric performance to construct a potential difference for ES and capacitive antibacterial capability at the wound site, thereby achieving comprehensive wound regulation from the inflammation-to-remodeling stage and promoting healthy tissue healing. This wound repair capability has never been reported in previous studies on intelligent sutures (Table S3). This represents a fundamental shift—from external energy coupling to internalized, therapeutic coupling—and opens new avenues for minimally invasive, self-powered medical devices.

Note S2. Body-coupled environmental electromagnetic energy

Compared with air ($\varepsilon \approx 1$ and $\sigma \approx 10^{-14}$ S/m, respectively), the human body has a very high relative permittivity and conductivity ($\varepsilon \approx 78$ and $\sigma \approx 0.6$ S/m, respectively), making it an ideal carrier for coupling electromagnetic energy[22]. When measured on the human body using electrodes, the observed power is about 30-40 dB more than when measured directly in air without the human body involved (Figure S1b). Although a wide spectrum of waves will couple to the human body, the 50 Hz wave (power line coupling) has the highest energy in the spectrum

due to the widespread use and prevalence of power grids and appliances. As a result, high-energy EM waves at 50 Hz will induce quasi-static-dynamic polarization in the body and store energy in the body. Due to the persistence of the EM waves, the stored energy is released and continues to light up the LEDs (Video S1).

Note S3. Generation and calculation of ES

Based on the circuit diagram of the electrical connection between the BET-suture, the human body, and the ambient electromagnetic wave source illustrated in Figure 2b, we can analyze the key parameters in the formation process of ES. The 50 Hz stimulating E-field is generated in the wound through the following steps: 1) the body-coupled ambient electromagnetic energy (V_b) is stored and transmitted in the body; 2) the transmitted energy induces dynamic polarization in the sheath layer of the BET-suture and generates a lagging induced potential (V_d) at the electrodes; 3) energy storage in the sheath layer will cause a potential difference ($V_{incision}$) to be created between V_b and V_d, resulting in ES. Due to the phase characteristics of AC circuits, the following vectorial relationship should exist between the three:

$$\overrightarrow{V_{incision}} = \overrightarrow{V_b} - \overrightarrow{V_d} \quad (3)$$

$$\left|\overrightarrow{V_{incision}}\right| = \sqrt{\left|\overrightarrow{V_b}\right|^2 - \left|\overrightarrow{V_d}\right|^2} \quad (4)$$

$V_{incision}$ is calculated directly from the phase angle (θ) between V_b and V_d:

$$tan\,\theta = \frac{X_d}{R_{tissue}} = \frac{1}{2\pi f C_d R_{tissue}} \quad (5)$$

$$\left|\overrightarrow{V_{incision}}\right| = \left|\overrightarrow{V_b}\right| sin\,\theta \quad (6)$$

Where, X_d and C_d represent the reactance and capacitance of the BET-suture dielectric layer, respectively; R_{tissue} is the resistance of the suturing tissue; and f is the frequency of the AC circuit, 50 Hz.

Based on the above equations, we can calculate the potential difference that generates the ES by measuring V_b and V_d in real time.

Note S4. Environmental parameters of ES

In order to accurately characterize the electrical performance of the BET-suture during the implementation of ES in the environment, we used the transmitter to simulate the

electromagnetic energy emitted in the environment and the corresponding frequency (23.8 dBm and 50 Hz, respectively). During ES performed by the BET-suture, the overall energy transfer circuit is established mainly through parasitic capacitance. This mainly consists of the parasitic capacitance between the transmitter and the body (C_b), the parasitic capacitance between the body and the BET-suture (C_d), and the parasitic capacitance between the body and the earth (C_p). All in vivo electrical signal data in this work were obtained in a closed room without any other electronics except for the transmitter and oscilloscope. Therefore, the intensity of the ES implemented by BET-suture was mainly affected by the distance from the electromagnetic source (Figure 2g).

Note S5. Size selection and advantages of Ag NPs

We measured the Zeta potential and particle size distribution of selected Ag NPs (Figure S11a and b). The Ag NPs were dispersed in deionized water and measured the Zeta potential and average particle size to be 9.44 eV and 199.27 nm, respectively. This indicated that the surface of Ag NPs carried a certain amount of Ag^+, giving it a positive charge. This positive potential surface would interact strongly with the negative surface of bacteria, causing cell membrane rupture and functional disorganization, further enhancing the capacitive antibacterial ability (Figure 3a).

We further selected Ag NPs with different particle sizes and Ag NWs as nano-doped particles for experimentation to validate the rationality of selecting the size of the Ag NPs. As shown in Figure S11c, under the same addition amount, PLGA/Ag NPs exhibited a higher relative permittivity than PLGA/Ag NWs. Meanwhile, the smaller the Ag NPs, the greater the improvement in dielectric properties. However, previous reports indicated that smaller Ag NPs have larger specific surface areas and stronger toxicity[23]. Especially when Ag NPs are in the tens of nanometres range, they exhibit significant toxicity. As shown in Figure S11d and e, at the same concentration (100 ppb), Ag NPs with an average particle size of 50 nm killed part of the cells. In contrast, Ag NPs and Ag NWs larger than 100 nm maintained good biocompatibility. Therefore, we selected Ag NPs with an average particle size of 200 nm to ensure treatment safety while maximizing ES capability.

In contrast, although Au NPs are stable, they lack antibacterial activity and are expensive;

while Zn and Mg NPs have certain biological activity, they are prone to oxidation and difficult to stabilize for enhancing the dielectric properties of polymers, making them unsuitable as functional nanofillers in this system.

Note S6. Differential analysis of recovery status and inflammation during in vivo incision therapy

The BET-suture group demonstrated the optimal recovery status, characterized by tissue color closest to normal, with no signs of redness, swelling, exudation, or necrosis. Healing was tight, with a smooth, continuous tissue surface, flat adhesion, and an appearance nearly identical to normal tissue, significantly superior to the Control, AB-suture, and BS-suture groups.

Staining of tissue sections after 7 days demonstrated a clear differentiation of inflammation (Figure 5c). In the control, AB-suture and ES-suture groups, the degree of inflammation (blue-violet color) gradually decreased, indicating that the unenergized BET-suture (AB-suture group) exhibited a certain degree of anti-inflammatory properties. In contrast, the unenergised BET-suture did not exhibit significant antibacterial and anti-inflammatory properties in the cellular experiments. The difference in the results could be attributed to the partial degradation of the sheath layer which released a certain amount of Ag NPs and produced antimicrobial and anti-inflammatory effects, thus achieving a better healing effect than the medical absorbable suture (control group). In addition, due to the presence of only trace amounts of Ag NPs, the effective ES provided by BET-suture achieved better anti-inflammatory capacity.

Note S7. Analysis of in vivo electrical performance changes

The evolution of the in vivo electrical properties came from the change in thickness due to the degradation of the sheath layer, which caused a change in the energy storage properties of this dielectric layer, and the trend was analyzed mainly by the following equations:

$$W_d = \frac{Q_b^2}{2C_d} \quad (7)$$

$$C_d = \frac{2\pi\varepsilon_d\varepsilon_0 r}{d} \quad (8)$$

Where, W_d is the energy stored in the dielectric layer, Q_b is the induced charge of the body-coupled electromagnetic energy, C_d is the capacitance of the dielectric layer, ε_d is the relative dielectric constant, ε_0 is the air dielectric constant, r is the radius of the suture, and d is the

thickness of the dielectric layer.

Under the condition of constant external electromagnetic field strength, the induced charge Q_b in Eq. (7) remains constant. Therefore, the energy storage capacity of the dielectric layer is inversely proportional to its capacitance. Meanwhile, the capacitance C_d is varied by the thickness of the dielectric layer and the radius of the suture. Since the thickness of the dielectric layer (15 μm) is thinner compared to the radius of the suture (33 μm), the ratio of r and d increases rapidly with a large change in d, resulting in a rapid decrease in the energy storage performance of the dielectric layer. Thus, Figure S24 showed that during the initial period of stitching (0-10 days, healing period), the electrical properties of BET-suture remained stable due to the fact that the sheath layer did not undergo significant degradation and the d change was small (Figure S5c). In contrast, after 14 days, the sheath layer began to undergo rapid degradation, d decreased rapidly, and the BET-suture electrical properties began to decrease dramatically and gradually lost ES.

Supplementary references

1. Sun, Z. et al. A bioabsorbable mechanoelectric fiber as electrical stimulation suture. *Nat. Commun.* **15**, 8462 (2024).
2. Wang, L. et al. Hierarchical helical carbon nanotube fibre as a bone-integrating anterior cruciate ligament replacement. *Nat. Nanotechnol.* **18**, 1085-1093 (2023).
3. Lee, J.S. et al. A multifunctional decellularized gut suture platform. *Matter* **6**, 2293-2311 (2023).
4. Cai, J. et al. Peptide-AIE Nanofibers Functionalized Sutures with Antimicrobial Activity and Subcutaneous Traceability. *Adv. Mater.* **36**, e2400531 (2024).
5. Lee, G. et al. A bioresorbable peripheral nerve stimulator for electronic pain block. *Sci. Adv.* **8**, eabp9169 (2022).
6. Bao, R. et al. Neuromorphic electro-stimulation based on atomically thin semiconductor for damage-free inflammation inhibition. *Nat. Commun.* **15**, 1327 (2024).
7. Wu, H. et al. Accelerated intestinal wound healing via dual electrostimulation from a soft and biodegradable electronic bandage. *Nat. Electron.* **7**, 299-312 (2024).
8. Ma, X. et al. A Mg Battery-Integrated Bioelectronic Patch Provides Efficient Electrochemical Stimulations for Wound Healing. *Adv. Mater.* **36**, e2410205 (2024).
9. Yao, G. et al. A programmable and skin temperature–activated electromechanical synergistic dressing for effective wound healing. *Sci. Adv.* **8**, eabl8379 (2022).
10. Jiang, Y. et al. Wireless, closed-loop, smart bandage with integrated sensors and stimulators for advanced wound care and accelerated healing. *Nat. Biotechnol.* **41**, 652–662 (2023).
11. Lee, D.-M. et al. An on-demand bioresorbable neurostimulator. *Nat. Commun.* **14**, 7315 (2023).
12. Meng, X. et al. An Ultrasound-Driven Bioadhesive Triboelectric Nanogenerator for Instant Wound Sealing and Electrically Accelerated Healing in Emergencies. *Adv. Mater.* **35**, e2209054 (2023).
13. Yao, G. et al. A self-powered implantable and bioresorbable electrostimulation device for biofeedback bone fracture healing. *PNAS* **118**, e2100772118 (2021).
14. Liu, Y. et al. Exercise-induced piezoelectric stimulation for cartilage regeneration in

rabbits. *Sci. Transl. Med.* **14**, eabi7282 (2022).
15. Xue, F. et al. Two way workable microchanneled hydrogel suture to diagnose, treat and monitor the infarcted heart. *Nat. Commun.* **15**, 864 (2024).
16. Liu, M. et al. Biomimicking Antibacterial Opto-Electro Sensing Sutures Made of Regenerated Silk Proteins. *Adv. Mater.* **33**, e2004733 (2021).
17. Ma, Z. et al. Bioinspired tough gel sheath for robust and versatile surface functionalization. *Sci. Adv.* **7**, eabc3012 (2021).
18. Wen, K. et al. Jellyfish-Inspired Artificial Spider Silk for Luminous Surgical Sutures. *Adv. Mater.* **36**, e2314158 (2024).
19. Li, J. et al. Body-coupled power transmission and energy harvesting. *Nat. Electron.* **4**, 530–538 (2021).
20. Yong, H. et al. Synchronous Generation of Electrical and Cellular Energies via Body-Mediated Energy Transfer: Inevitable Electric Field Concentration. *ACS Energy Lett.* **8**, 2954-2961 (2023).
21. Kang, M. et al. Self-Powered Electrical Bandage Based on Body-Coupled Energy Harvesting. *Adv. Mater.* **36**, 2402491 (2024).
22. Yang, W. et al. Single body-coupled fiber enables chipless textile electronics. *Science* **384**,74–81 (2024).
23. Janzadeh, A. et al. The Toxic Effect of Silver Nanoparticles on Nerve Cells: A Systematic Review and Meta-Analysis. *RECT* **257**, 93-119 (2021).
