# Supplementary material for: A bioabsorbable body-coupling-electrotherapy suture
Source: Nat Commun. 2025 Dec 10;16:11156. doi: 10.1038/s41467-025-66045-8 (PMC12708654; doi:10.1038/s41467-025-66045-8)
Supplement: Supplementary file 2 — Description of Additional Supplementary Files [file 41467_2025_66045_MOESM2_ESM.pdf]

Description of Additional supplementary Files

Supplementary Movie 1 Body-coupled ambient electromagnetic energy to light up LEDs;
Supplementary Movie 2 Coupling voltage of the human body in different movement states;
Supplementary Movie 3 BET-suture maintaining stable electrical properties in vivo.
