# Supplementary material for: A bioabsorbable body-coupling-electrotherapy suture
Source: Nat Commun. 2025 Dec 10;16:11156. doi: 10.1038/s41467-025-66045-8 (PMC12708654; doi:10.1038/s41467-025-66045-8)
Supplement: Supplementary file 6 — Reporting Summary [file 41467_2025_66045_MOESM6_ESM.pdf]

nature portfolio

Corresponding author(s): Chengyi Hou

Last updated by author(s): October 1, 2025

# Reporting Summary

Nature Portfolio wishes to improve the reproducibility of the work that we publish. This form provides structure for consistency and transparency in reporting. For further information on Nature Portfolio policies, see our Editorial Policies and the Editorial Policy Checklist.

## Statistics

For all statistical analyses, confirm that the following items are present in the figure legend, table legend, main text, or Methods section.

| n/a | Confirmed | |
|---|---|---|
| ☐ | ☒ | The exact sample size (*n*) for each experimental group/condition, given as a discrete number and unit of measurement |
| ☐ | ☒ | A statement on whether measurements were taken from distinct samples or whether the same sample was measured repeatedly |
| ☐ | ☒ | The statistical test(s) used AND whether they are one- or two-sided<br>*Only common tests should be described solely by name; describe more complex techniques in the Methods section.* |
| ☒ | ☐ | A description of all covariates tested |
| ☒ | ☐ | A description of any assumptions or corrections, such as tests of normality and adjustment for multiple comparisons |
| ☐ | ☒ | A full description of the statistical parameters including central tendency (e.g. means) or other basic estimates (e.g. regression coefficient) AND variation (e.g. standard deviation) or associated estimates of uncertainty (e.g. confidence intervals) |
| ☐ | ☒ | For null hypothesis testing, the test statistic (e.g. *F*, *t*, *r*) with confidence intervals, effect sizes, degrees of freedom and *P* value noted<br>*Give P values as exact values whenever suitable.* |
| ☒ | ☐ | For Bayesian analysis, information on the choice of priors and Markov chain Monte Carlo settings |
| ☒ | ☐ | For hierarchical and complex designs, identification of the appropriate level for tests and full reporting of outcomes |
| ☒ | ☐ | Estimates of effect sizes (e.g. Cohen's *d*, Pearson's *r*), indicating how they were calculated |

*Our web collection on statistics for biologists contains articles on many of the points above.*

## Software and code

Policy information about availability of computer code

| | |
|---|---|
| Data collection | Keithley 6514E and oscilloscope were employed to test the electrical output performance of BET-suture in vitro and in vivo. The electromyography (EMG) signals were recorded with time by Neusen.U16. |
| Data analysis | The statistical difference among different groups was accessed via one-way analysis of variance (ANOVA) methods. Image J, Origin 2018b and Excel were used for data analysis and plotting. |

For manuscripts utilizing custom algorithms or software that are central to the research but not yet described in published literature, software must be made available to editors and reviewers. We strongly encourage code deposition in a community repository (e.g. GitHub). See the Nature Portfolio guidelines for submitting code & software for further information.

## Data

Policy information about availability of data

All manuscripts must include a data availability statement. This statement should provide the following information, where applicable:

- Accession codes, unique identifiers, or web links for publicly available datasets
- A description of any restrictions on data availability
- For clinical datasets or third party data, please ensure that the statement adheres to our policy

All data supporting the findings of this study are available within the article and its supplementary files.

## Research involving human participants, their data, or biological material

Policy information about studies with human participants or human data. See also policy information about sex, gender (identity/presentation), and sexual orientation and race, ethnicity and racism.

| | |
|---|---|
| Reporting on sex and gender | No gender-based analysis was conducted in this study. |
| Reporting on race, ethnicity, or other socially relevant groupings | The study focused on other aspects, and no information related to race, ethnicity, or other socially relevant groupings was collected or analyzed. As a result, there were no confounding variables related to these factors that needed to be controlled in our analyses. |
| Population characteristics | No population characteristics was conducted in this study. |
| Recruitment | The participants were recruited internally from within the research group. We provided a detailed description of the study, explicitly stating that participation was voluntary and non-participation would have no impact. |
| Ethics oversight | All animal experiments were performed according to protocols approved by approved by Committee on Ethics of Donghua University (DHUEC-NSFC-2022-43). |

Note that full information on the approval of the study protocol must also be provided in the manuscript.

# Field-specific reporting

Please select the one below that is the best fit for your research. If you are not sure, read the appropriate sections before making your selection.

☒ Life sciences ☐ Behavioural & social sciences ☐ Ecological, evolutionary & environmental sciences

For a reference copy of the document with all sections, see nature.com/documents/nr-reporting-summary-flat.pdf

# Life sciences study design

All studies must disclose on these points even when the disclosure is negative.

| | |
|---|---|
| Sample size | Six-eight-weeks-old Sprague-Dawley female rats with an average weight of 250–300 g were kept in a certified animal facility. The dataset includes one video segments and multiple images. |
| Data exclusions | No data excluded |
| Replication | Different devices made with the same materials were tested for multiple times with different activities. |
| Randomization | Randomization was not relevant to our study since experiments were specific to each subject. |
| Blinding | Randomization was not relevant to our study since experiments were specific to each subject. |

# Reporting for specific materials, systems and methods

We require information from authors about some types of materials, experimental systems and methods used in many studies. Here, indicate whether each material, system or method listed is relevant to your study. If you are not sure if a list item applies to your research, read the appropriate section before selecting a response.

### Materials & experimental systems

| n/a | Involved in the study | |
|---|---|---|
| ☐ | ☒ | Antibodies |
| ☐ | ☒ | Eukaryotic cell lines |
| ☒ | ☐ | Palaeontology and archaeology |
| ☐ | ☒ | Animals and other organisms |
| ☒ | ☐ | Clinical data |
| ☒ | ☐ | Dual use research of concern |
| ☒ | ☐ | Plants |

### Methods

| n/a | Involved in the study | |
|---|---|---|
| ☒ | ☐ | ChIP-seq |
| ☒ | ☐ | Flow cytometry |
| ☒ | ☐ | MRI-based neuroimaging |

## Antibodies

| | |
|---|---|
| Antibodies used | Rabbit anti-Ki-67, p-PI3K, PI3K, p-AKT, AKT, p-ERK, ERK, CCND1, p-CDC2, β-actin antibodies |

| | |
|---|---|
| Validation | Rabbit anti-Ki-67 antibody was purchased from Abcam and used to detect the proliferation of fibroblasts under electrical stimulation. All other antibodies were validated by the manufacturers for Western blotting and are widely used in published literature. |

## Eukaryotic cell lines

Policy information about cell lines and Sex and Gender in Research

| | |
|---|---|
| Cell line source(s) | L929 fibroblasts are derived from Chinese Academy of Science Cell Bank |
| Authentication | None of the cell lines used were authenticated. |
| Mycoplasma contamination | The cell lines were not tested for mycoplasma contamination. |
| Commonly misidentified lines (See ICLAC register) | None of commonly misidentified cell lines used in the study. |

## Animals and other research organisms

Policy information about studies involving animals; ARRIVE guidelines recommended for reporting animal research, and Sex and Gender in Research

| | |
|---|---|
| Laboratory animals | Six-eight-weeks-old Sprague-Dawley female rats with an average weight of 250–300 g were kept in a certified animal facility. |
| Wild animals | No wild animals were used in the study. |
| Reporting on sex | No reporting on sex in the study. |
| Field-collected samples | No field-collected samples on sex in the study. |
| Ethics oversight | All animal experiments were performed according to protocols approved by approved by Committee on Ethics of Donghua University (DHUEC-NSFC-2022-43). |

Note that full information on the approval of the study protocol must also be provided in the manuscript.

## Plants

| | |
|---|---|
| Seed stocks | *Report on the source of all seed stocks or other plant material used. If applicable, state the seed stock centre and catalogue number. If plant specimens were collected from the field, describe the collection location, date and sampling procedures.* |
| Novel plant genotypes | *Describe the methods by which all novel plant genotypes were produced. This includes those generated by transgenic approaches, gene editing, chemical/radiation-based mutagenesis and hybridization. For transgenic lines, describe the transformation method, the number of independent lines analyzed and the generation upon which experiments were performed. For gene-edited lines, describe the editor used, the endogenous sequence targeted for editing, the targeting guide RNA sequence (if applicable) and how the editor was applied.* |
| Authentication | *Describe any authentication procedures for each seed stock used or novel genotype generated. Describe any experiments used to assess the effect of a mutation and, where applicable, how potential secondary effects (e.g. second site T-DNA insertions, mosiacism, off-target gene editing) were examined.* |
